# Heterogeneity in the projections and excitability of tyraminergic/octopaminergic neurons that innervate the *Drosophila* reproductive tract

**DOI:** 10.3389/fnmol.2024.1374896

**Published:** 2024-08-02

**Authors:** Ethan W. Rohrbach, James D. Asuncion, Pratap Meera, Mason Kralovec, Sonali A. Deshpande, Felix E. Schweizer, David E. Krantz

**Affiliations:** ^1^Interdepartmental Program in Neuroscience, David Geffen School of Medicine at UCLA, Los Angeles, CA, United States; ^2^Medical Scientist Training Program, David Geffen School of Medicine at UCLA, Los Angeles, CA, United States; ^3^Department of Neurobiology, David Geffen School of Medicine at UCLA, Los Angeles, CA, United States; ^4^UCLA College of Arts and Sciences, Los Angeles, CA, United States; ^5^Department of Psychiatry and Biobehavioral Sciences, Hatos Center for Neuropharmacology, Gonda (Goldschmied) Neuroscience and Genetics Research Center, David Geffen School of Medicine at UCLA, Los Angeles, CA, United States

**Keywords:** ventral nerve cord, ovulation, oviposition, adrenergic, neuromodulation, octopamine, serotonin, monoamines

## Abstract

Aminergic nuclei in mammals are generally composed of relatively small numbers of cells with broad projection patterns. Despite the gross similarity of many individual neurons, recent transcriptomic, anatomic and behavioral studies suggest previously unsuspected diversity. Smaller clusters of aminergic neurons in the model organism *Drosophila melanogaster* provide an opportunity to explore the ramifications of neuronal diversity at the level of individual cells. A group of approximately 10 tyraminergic/octopaminergic neurons innervates the female reproductive tract in flies and has been proposed to regulate multiple activities required for fertility. The projection patterns of individual neurons within the cluster are not known and it remains unclear whether they are functionally heterogenous. Using a single cell labeling technique, we show that each region of the reproductive tract is innervated by a distinct subset of tyraminergic/octopaminergic cells. Optogenetic activation of one subset stimulates oviduct contractions, indicating that the cluster as a whole is not required for this activity, and underscoring the potential for functional diversity across individual cells. Using whole cell patch clamp, we show that two adjacent and morphologically similar cells are tonically inhibited, but each responds differently to injection of current or activation of the inhibitory GluCl receptor. GluCl appears to be expressed at relatively low levels in tyraminergic/octopaminergic neurons within the cluster, suggesting that it may regulate their excitability via indirect pathways. Together, our data indicate that specific tyraminergic/octopaminergic cells within a relatively homogenous cluster have heterogenous properties and provide a platform for further studies to determine the function of each cell.

## Introduction

Aminergic nuclei such as the locus coeruleus and the raphe contain multiple neurons that release the same aminergic neurotransmitter and project to similar targets (Andrade and Haj-Dahmane, 2013; Okaty et al., 2020; Poe et al., 2020). In some cases, anatomical, molecular or functional differences can distinguish similar subsets of cells within these nuclei (Andrade and Haj-Dahmane, 2013; Soiza-Reilly and Commons, 2014; Chandler et al., 2019; Huang et al., 2019; Borodovitsyna et al., 2020). Markers for many other subtypes of aminergic neurons are lacking, making it difficult to identify them or determine their post-synaptic targets. In addition, since many aminergic neurons use volume rather than synaptic transmission, standard molecular tools that use synaptic markers to map connectivity are often unapplicable (Fuxe et al., 2010). As a result, the potential heterogeneities between many aminergic neurons remain poorly understood.

Aminergic nuclei in mammals contain thousands or millions of neurons depending on the species, thereby complicating the identification of specific subtypes. In addition, the location of specific cells within the nuclei are not precisely fixed. By contrast, the relatively small number of neurons and their stereotyped location in invertebrates has greatly facilitated the analysis of their function, as evidenced by classical studies in *C*. *elegans*, crab, lobster, and locust (Kravitz and Huber, 2003; Lange, 2009; Bargmann and Marder, 2013). We are similarly using *Drosophila melanogaster* to analyze the projections and functional properties of an aminergic cluster in the ventral nerve cord of the female fly.

Octopamine release is required for sperm storage, egg maturation, and contractility of the oviducts in flies as well as other insects (Kalogianni and Theophilidis, 1993; Monastirioti et al., 1995; Clark and Lange, 2003; Monastirioti, 2003; Middleton et al., 2006; Rodriguez-Valentin et al., 2006; Lange, 2009; Avila et al., 2012; Rezaval et al., 2014; Meiselman et al., 2018; Pauls et al., 2018; Hana and Lange, 2020; Yoshinari et al., 2020; White et al., 2021). In *Drosophila*, these processes are associated with a cluster of neurons in the abdominal ganglion of the ventral nerve cord that innervate the female reproductive tract (Monastirioti et al., 1995; Monastirioti, 2003; Rodriguez-Valentin et al., 2006; Rezaval et al., 2012, 2014; Pauls et al., 2018; White et al., 2021). Since it is possible that these neurons also release tyramine we refer to them as tyraminergic/octopaminergic. We also describe these cells here as the “posterior cluster” to draw a distinction between them and other, more anterior neurons that target the body wall or other sites (Monastirioti et al., 1995; Monastirioti, 2003; Rodriguez-Valentin et al., 2006; Rezaval et al., 2014; Pauls et al., 2018; White et al., 2021). To our knowledge, no other octopaminergic neurons elsewhere in the ventral nerve cord have been proposed to regulate egg-laying.

Similar to other midline, unpaired neurons in insects, each one of these cells extends a single, large process that branches into two bilaterally symmetric extensions (Hoyle et al., 1980; Kalogianni and Theophilidis, 1993; Horner, 1999). The specific targets in the reproductive tract innervated by each cell in the abdominal ganglion and the potential differences between their physiology and function are not known. Identifying their targets and physiological properties would represent a key step toward understanding the function and regulation of the oviposition circuit, complementing previous studies that have identified both peripheral and central regulatory pathways (Lee et al., 2003, 2009; Hasemeyer et al., 2009; Castellanos et al., 2013; Rubinstein and Wolfner, 2013; Gou et al., 2014; Heifetz et al., 2014; Lim et al., 2014; Meiselman et al., 2018; Wang et al., 2020a,b).

Using a single cell labeling technique (Nern et al., 2015), we show that each neuron within the cluster at the tip of the abdominal ganglion targets a distinct region of the reproductive tract. Optogenetic stimulation of a small subset of these cells drives lateral oviduct contractions, consistent with the idea that *en bloc* activity of the cluster is not required for this activity, and that each cell may serve a distinct function. Patch clamp recordings of two adjacent neurons show differences in electrophysiological excitability and inhibitory regulation. These data establish a framework for studying the role of different aminergic neurons within an anatomically defined cluster, and how each may contribute to the function of the cluster as a whole.

## Materials and methods

### Multi-color flip out and immunohistochemistry

To map the projection targets of individual Tdc2(+) neurons, Multi-Color Flip Out (MCFO) experiments were carried out using *Tdc2-Gal4* (Cole et al., 2005) and *UAS-MCFO7* (Nern et al., 2015). Flies were aged to 7–10 days post eclosion, and the reproductive systems with the adjoined central nervous system was dissected in phosphate buffered saline (PBS, 11.8 mM Na/K-P_i_, 137 mM NaCl, PH. 7.4, ThermoFisher). The tissue was fixed in 4% paraformaldehyde, washed 3× in PBS + 0.3% TritonX100 and blocked in 5% normal goat serum (Sigma-Aldrich) in PBS + 0.3% TritonX100 at room temperature followed by incubation in primary antibodies against V5, HA, and FLAG epitopes (Mouse-anti-V5, 1/500, ThermoFisher; Rabbit-anti-ΗA, 1/300, Cell Signaling Technology; Rat-anti-FLAG, 1/200, Novus Biologicals) overnight at 4°C, and secondary antibodies (Goat anti-Mouse-AlexaFluor488, ThermoFisher; Goat anti-Rabbit-AlexaFluor555, 1/500, ThermoFisher; Goat anti-Rat-AlexaFluor633, 1/500, ThermoFisher) for 3 h at room temperature. The preparations were washed in PBS + 0.3% TritonX100 then cleared in 25% glycerol in PBS overnight at 4°C and mounted in Fluoromount-G (Southern Biotech) under a cover slip (#0, Electron Microscopy Sciences) raised ~100 μm as a “bridge” between two additional coverslips to reduce compression of the tissue. Tissue was imaged using a Zeiss LSM 880 confocal microscope. Images of the reproductive tract and VNC were obtained at 1 μm and 500 nm intervals, respectively, and analyzed using ImageJ software. A total of 58 individual preparations were analyzed.

Co-labeling of muscle and tyraminergic/octopaminergic neurons was performed using *Tdc2-Gal4* to express *UAS::mCD8-GFP* followed by mouse-anti-GFP (1/500, Sigma-Aldrich) and AF555-conjugated phalloidin. Co-expression between the drivers *Tdc2-LexA* and either *J39942-Gal4* or *GluCl-Gal4* was performed using the reporters *UAS::mCD8-GFP* and *LexAop::CD2-RFP*. Preparations were dissected, fixed, labeled, and imaged as described above for MCFO. Primary antibodies against GFP and RFP epitopes (Mouse-anti-GFP, 1/500, Sigma-Aldrich; Rabbit anti-dsRED, 1/500, Takara Bio) were used with the secondary antibodies (Goat anti-Mouse-488, 1/500, ThermoFisher; Goat anti-Rabbit-AlexaFluor 555, 1/500, ThermoFisher) and 4′,6-Diamidino-2-phenylindole dihydrochloride (DAPI, 1/1000, Sigma-Aldrich). Antibodies, genetic stocks, and other reagents are summarized in Supplementary Table T2.

### Optogenetics and lateral oviduct contraction assay

Optogenetic stimulation was performed using either *Tdc2-Gal4* or a Gal4 line targeting the *tyramine β hydroxylase* gene (*J399342-Gal4*) to express *UAS-ChR2-XXM::tdTomato*. Flies harboring one copy of *J399342-Gal4* and one copy of *UAS-ChR2-XXM::tdTomato* were compared to control flies with one copy of *Tdc2-Gal4* and *UAS-ChR2-XXM::tdTomato* (positive control) or one copy of *UAS-ChR2-XXM::tdTomato* alone (negative control). Flies were dissected on a Sylgard disk in HL3.1 solution (pH = 7.3; 70 mM NaCl, 5 mM KCl, 5 mM trehalose, 2 mM CaCl2, 4 mM MgCl2, 115 mM sucrose, 10 mM NaHCO3) (Feng et al., 2004). The legs and wings were removed, and the fly was immobilized with the ventral side facing up using one insect pin around the cervical connective to secure the head and another pin placed through the most posterior region of dorsal cuticle to secure the abdomen. Using sharp forceps, the ventral cuticle of the metathorax and two abdominal sternites were removed to expose the abdominal ganglion and lateral oviducts, respectively. The anterior sternites of the abdomen between the two dissected windows were left in place. Optogenetic stimulation and imaging were performed using a Zeiss Axio Examiner Z1 system equipped with two ThorLabs LEDs (M565L3 and M470L4 with Thorlabs drivers LEDD1B and DC2200 respectively), a custom beam combiner (Thorlabs) and an Andor iXon X3 camera (Oxford Instruments). To stimulate *ChR2-XXM* at ~470 nm and visualize the prep with excitation at ~565 nm, we used a custom filter set that included a dual band excitation filter with peaks at 484 and 561 (FF01-484/561), a 593 nm high pass dichroic (FF593-Di03), and the single band emission filter (FF01-620/52). Stimulation was initiated and stopped by manually turning the DC2200/M470L4-C4 LED on and off. The intensity of the ~470 nm illumination within the field of view was determined to be 1 mW/mm^2^ using a Thorlabs digital handheld optical power meter. Lateral oviduct contractions were manually counted in video recordings, with contraction times noted at the time of maximal contraction. Contractions were defined by a decrease in the distance between ovaries and a characteristic contraction of the oviduct tissue. These movements can be distinguished from random movements of the prep in either the *x*-*y* plane or the *z* axis or from contractions of the ovaries.

### Electrophysiology

Electrophysiological recordings were performed in flies harboring one copy each of *Tdc2-Gal4* and *UAS::mCD8-GFP*. Flies aged 4–8 days post eclosion were anesthetized on ice and dissected ventral side up on a Sylgard disk in HL3.1 solution (Feng et al., 2004) as described for optogenetic experiments. A pipette filled with 1% Protease IV solution was briefly “puffed” to remove the glial sheath and to expose the neurons for patching. GFP fluorescence was used to visualize the neurons, and whole-cell patch-clamp recordings from PC1 or PC2 were obtained with either continuous HL3.1 perfusion or perfusion of HL3.1 containing the indicated concentration of picrotoxin or ivermectin (Sigma Aldrich). A borosilicate glass pipette filled with internal solution containing 140 mM potassium aspartate, 10 mM HEPES, 1 mM KCl, 4 mM Mg-ATP, 0.5 mM Na_3_GTP, 1 mM EGTA (pH 7.3) and a resistance of ~7 MegaOhm was used for recordings. A series of step current injections were applied to the cell to elicit action potentials using a pClamp program with Multiclamp 700B amplifier, filtered at 4 kHz and sampled at 10 kHz with a Digidata 1300b (Molecular Devices). The response to injected current is reported as the number of action potentials or normalized response (action potentials/maximum response) as indicated. For normalization, the maximum number of action potentials elicited with current injection in a given cell was set to 1 and all other action potentials were divided by this number. During a subset of recordings, patched cells were filled with biocytin dye. Following recordings, these preparations were dissected in cold PBS, then fixed and labeled as described above using a primary antibody to GFP (Mouse-anti-GFP) followed by anti-Mouse-488 and co-labeling with streptavidin-555.

### Statistical analysis

For optogenetic experiments, the initial comparison of the negative control vs. the two experimental lines used non-parametric Kruskal-Wallis test with multiple comparisons in the program Prism (Graphpad). Subsequent comparison of the two experimental lines used the non-parametric Mann–Whitney test in Prism. For the electrophysiological experiments, the analysis was done using regression analysis in *R* with the function *lm*. For data collected in the absence of additional drugs, the equation used was *y* = β_0_ + β_1_*x*_1_ + β_2_*x*_2_ + ε in which *y* is the number of action potentials, β_0_ is the coefficient for the intercept, *x*_1_ is the current being applied, β_1_ is the coefficient for the current, *x*_2_ is a dummy variable corresponding to whether the observation came from PC1 or PC2 (0 for PC1, 1 for PC2), β_2_ is the coefficient for the effects of the cell type and ε is the unobservable error term. For the data collected in the presence of additional drugs, the equation used was *y* = β_0_ + β_1_*x*_1_ + β_2_*x*_2_ + β_3_*x*_3_ + ε. y, β_0_, β_1_, *x*_1_, and ε were as above, with *x*_2_ and *x*_3_ corresponding to whether or not treatment with ivermectin+PTx or PTx alone was applied, and β_2_ and β_3_ representing the coefficients for the effects of each treatment. The *R* function *summary* was applied to provide the estimate of the coefficients and the associated *p* value. The results are summarized in Supplementary Table T1.

## Results

### Individual tyraminergic/octopaminergic neurons that innervate the reproductive tract have different targets

**Figure 1:**
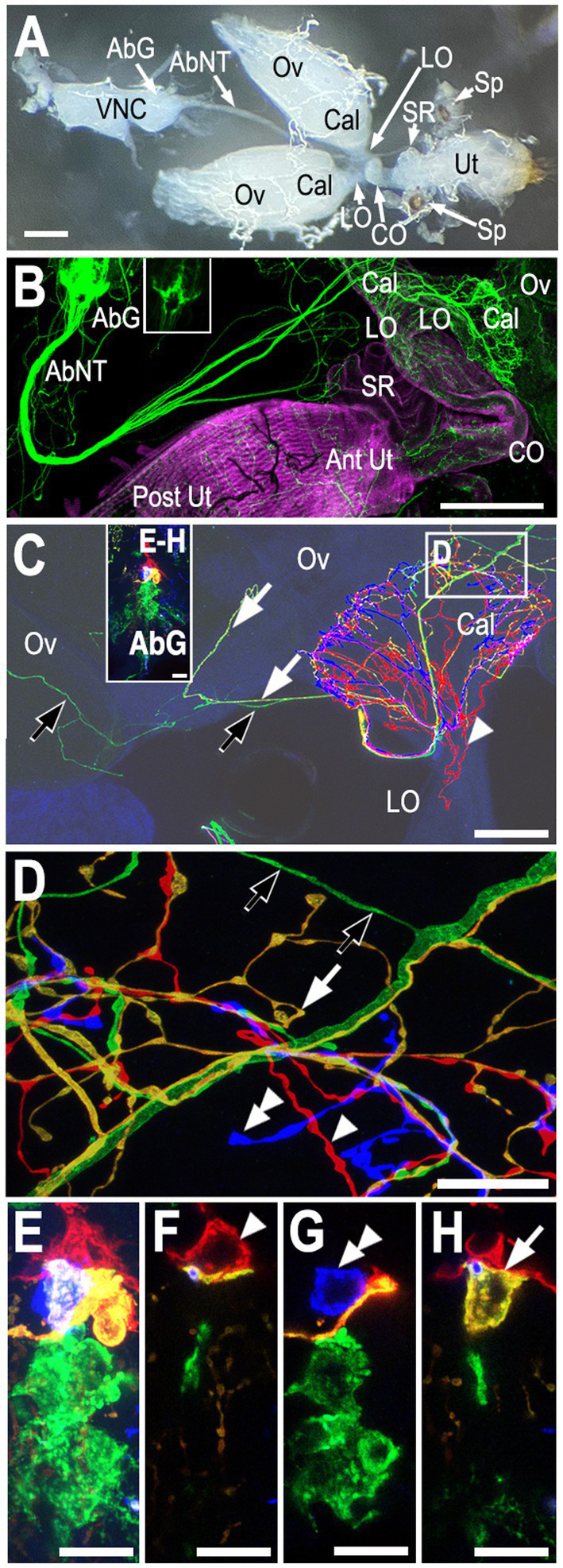
Overview of the reproductive tract and innervation of the calyx. **(A)** A light micrograph of the female reproductive tract and the ventral nerve cord (VNC) attached via the by the abdominal nerve trunk (Court et al., 2020) (aka the median abdominal nerve or MAN; Power, 1948). The ovaries (Ov), calyx (Cal), lateral oviducts (LO), common oviduct (CO), uterus (Ut), seminal receptacle (SR), and spermathecae (Sp) are indicated. **(B)** Co-labeling of *Tdc2-Gal4*(+) neurons with *UAS::mCD8-GFP* (green) and muscle with fluorophore-conjugated phalloidin (magenta). Both the anterior uterus (Ant Ut) and posterior uterus (Post Ut) are indicated. The spermathecae are not visible. Labels are otherwise as in panel **(A)**. Note that the image in panel **(B)** has been overexposed to allow visualization of fine processes; the inset in panel **(B)** shows an image of the VNC without over-exposure. **(C)** A confocal stack showing an MCFO-labeled reproductive tract. The abdominal ganglion (AbG) is shown in panels **(E–H)** and as an inset in panel **(C)**. The ovary (Ov), calyx (Cal), and lateral oviduct (LO) are indicated. Green (black arrows), yellow (white arrows), and red (white arrowhead) processes are also indicated. **(D)** A single optical slice of the boxed region of the calyx in panel **(C)** shown at higher resolution. The yellow (white arrow), red (white arrowhead), and blue (double white arrowhead) are derived from the correspondingly indicated cell bodies in the abdominal ganglion shown in panels **(E–H)** (and in the panel **C** inset). The projections of the yellow, red, and blue cells are represented as PC8, PC6, and PC7 in Figure 4A. The origin of the green processes cannot be determined from this preparation alone since there is more than one green cell body but, based on other labelings, are derived from the cell indicated as PC4 in Figure 4. Panels **(E–H)** A confocal stack (**E**, see also panel **C** inset) and single optical slices **(F–H)** of the abdominal ganglion showing one red cell body (white single arrowhead, indicated as PC6 in Figures 4B,C), one blue (white double arrowhead, PC7 in Figures 4B,C), and one yellow cell (white arrow, PC8 in Figures 4B,C) plus at least two more posterior green cells. Scale bars: **(A–B)** 100 μm. **(C)** 50 μm. **(D–H)** 10 μm.

The female reproductive tract of *Drosophila* includes the ovaries, calyx, lateral oviduct, common oviduct, uterus, seminal receptacle, and spermatheca (Figure 1A), plus the parovarian glands (not shown). Projections from the abdominal ganglion in the posterior-most region of the ventral nerve cord innervate the reproductive tract via the abdominal nerve trunk (Figures 1A,B) (Pauls et al., 2018; Court et al., 2020) also known as the median abdominal nerve (Power, 1948). A cluster of tyraminergic/octopaminergic neurons that localize to this area broadly innervate the reproductive tract (Figure 1B) (Monastirioti et al., 1995; Monastirioti, 2003; Rodriguez-Valentin et al., 2006; Schneider et al., 2012; Rezaval et al., 2014). We have designated these neurons as the “posterior cluster” to differentiate them from more anterior Tdc2(+) neurons in the abdominal ganglion (Schneider et al., 2012).

To map the projections of Tdc2(+) neurons that innervate the reproductive tract we used the single-cell labeling technique Multi-Color Flip Out (MCFO) (Nern et al., 2015). In brief, expression of three transgenes with different molecular tags allowed labeling of individual cells with three different fluorophores; expression is limited to one subtype of neurons using the Gal4/UAS system (Nern et al., 2015). Stochastic recombination of the tagged transgenes restricted labeling to a relatively small number of cells that express the Gal4 driver, with each combination of tags generating a distinct color. In an attempt to exclude tyraminergic cells and more specifically label octopaminergic neurons, we first tested a tyramine β hydroxylase Gal4 driver (Schneider et al., 2012). Unfortunately, although it specifically labeled octopaminergic neurons in the brain (Schneider et al., 2012), it did not label the octopaminergic neurons in the cluster of cells that innervates that reproductive tract (data not shown). We therefore used *Tdc2-Gal4* (Cole et al., 2005) to express MCFO in cells that synthesize both octopamine and tyramine and those that synthesize tyramine alone, but confined our analysis to midline neurons which are likely to synthesize both octopamine and tyramine and have been labeled as octopaminergic in previous anatomic analyses (Monastirioti et al., 1995; Monastirioti, 2003).

To map the projections of the Tdc2(+) cells in the labeled preparations, we first determined whether any processes in the reproductive tract were immunolabeled with MCFO, then determined which cell(s) in the nerve cord corresponded to the color that we observed in the reproductive tract. In some cases, projections in the reproductive tract could be unambiguously assigned to an individual cell. When more than one set of processes and/or cells were identically labeled, comparison of data from several experiments allowed us to deduce their identity. We did not detect any processes that labeled the reproductive tract and mapped to a region outside of the posterior tip of the abdominal ganglion, and all identified cells in this “posterior cluster” projected to the reproductive tract through the median abdominal nerve.

We detected three distinctly labeled arborizations innervating the calyx (Figures 1C,D), which correspond to three different cell bodies in the posterior cluster (PC) (Figures 1E–H). These include one cell body labeled red in the preparation shown in Figure 1 (Figure 1F, single white arrowhead) that projected into the calyx as well as the lateral oviducts (Figures 1C,D, single white arrowheads) which we have designated PC6. An adjacent, yellow cell that innervated the calyx also sent a small number of projections into the ovary, one of which can be visualized here (Figures 1C,D white arrows, PC8). The arborizations of an another, uniquely identified blue cell in this preparation (Figure 1G, double white arrowhead, PC7) appeared to be confined to the calyx (Figure 1C). Additional green processes were present in the uterus (not shown) and also passed through the calyx to innervate areas in the ovary more distal from the calyx (Figures 1C,D, black arrows). Since at least two cells in the posterior cluster were labeled green it was not possible to determine which one projected to the uterus vs. the ovaries in this preparation.

**Figure 2:**
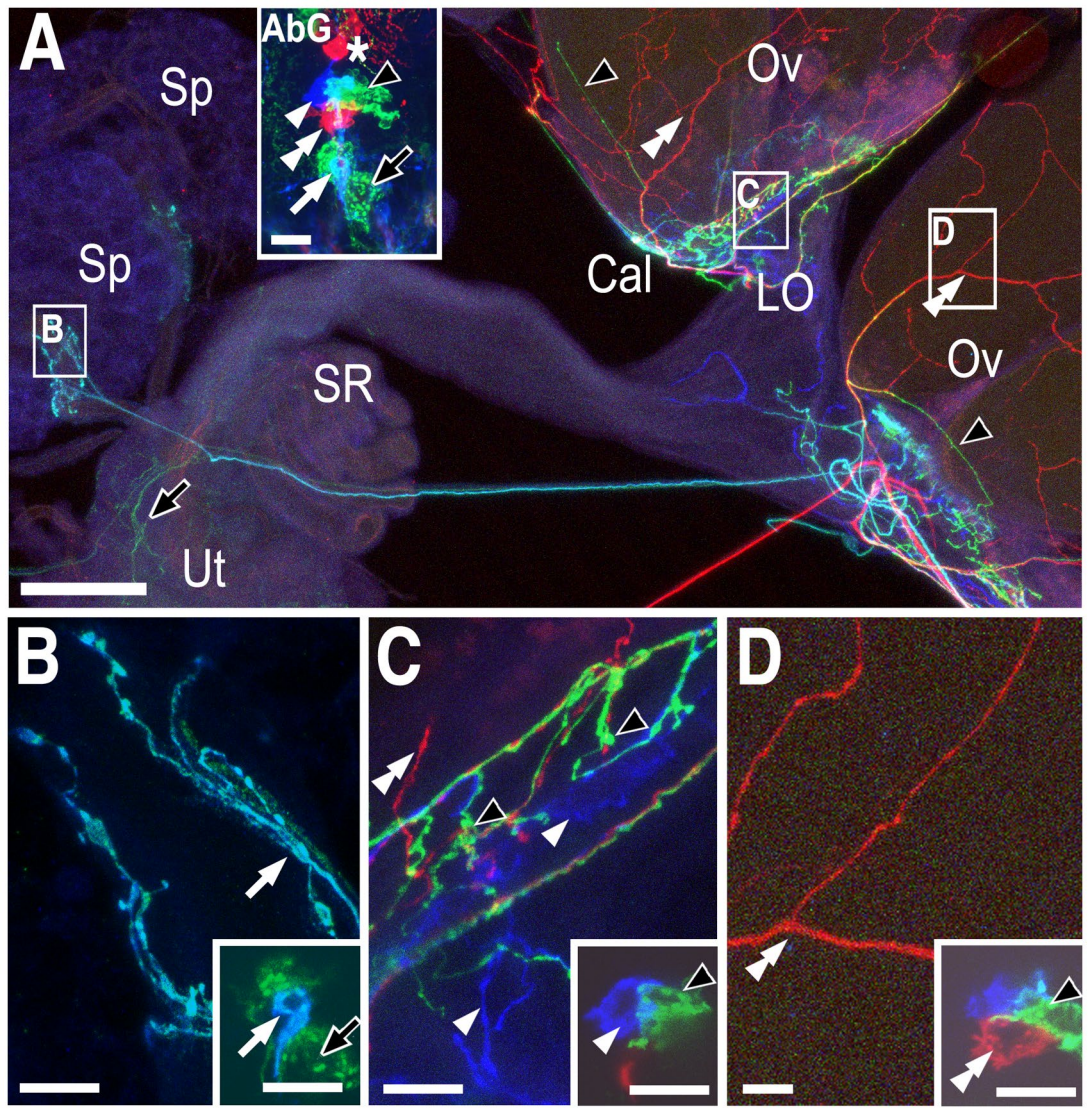
Neurons innervating the calyx, lateral oviducts, ovaries, and spermathecae. **(A)** Low magnification view of the labeled reproductive tract with the spermathecae (Sp), calyx (Cal), lateral oviducts (LO) ovaries (Ov), seminal receptacle (SR), and uterus (Ut) indicated. The inset shows labeled cells in the attached abdominal ganglion (AbG). Red processes in the ovaries (white double arrowheads), turquoise processes in the spermathecae (white arrow), and green processes in the both the uterus (black arrow) and the base of the ovaries (black arrowheads) are indicated. The inset in panel **(A)** shows one red cell that projects into the anterior neuropil (asterisk), and a second red cell (white double arrowhead) within the posterior cluster represented as PC4 in Figure 4. A single blue cell (single white arrowhead), a single turquoise cell (white arrow) and at least two green cells (black arrowhead and black arrow) are also visible. Panels **(B–D)** correspond to the boxed areas of the reproductive tract shown in panel **(A)**. Insets represent single optical slices from the confocal stack of the AbG shown in the inset in panel **(A)**. **(B)** The small turquoise cell (inset white arrow) innervates the spermathecae (white arrow) and is indicated as SpB in Figure 4. Based on other labelings, the large green cell (black arrow in inset) is PC3 and projects to the uterus (black arrow in **(A)**). **(C)** The blue cell (inset, single white arrowhead) innervates the calyx and the lateral oviducts (white arrowheads) and is shown as PC6 in Figure 4. Based on other labelings, the adjacent green cell (inset, black arrowhead) is PC8 and innervates the calyx with a few processes projecting into the base of the ovaries (black arrowheads in panels **(A**,**C)**). The overlap between the blue PC6 cell and the green PC8 cell appears turquoise (see panel **(A)** inset) but does not represent a distinct cell body. **(D)** The red cell is PC4 and innervates the ovaries (white double arrowheads). Scale bars: **(A)** 50 μm. **(B–D)** and insets in **(A–D)**: 10 μm.

In another preparation, red processes that innervated the ovaries (Figures 2A,D, double white arrowheads) could be matched to a single red cell within the posterior cluster (Figure 2A inset, Figure 2D inset, double white arrowheads, PC4). A relatively small turquoise cell (Figure 2A inset, Figure 2B inset, white arrow) innervated the stalk of the spermathecae (Figures 2A,B, white arrow) in the same preparation and is designated SpB. We identified another small cell at the anterior tip of the cluster that also innervated the spermathecae (orange cell in Supplementary Figure S1B and inset within Supplementary Figure S1C) and we have designated this cell as SpA. Based on other labelings, the green processes in the calyx and ovary (Figure 2C, black arrowhead, equivalent to PC8 in Figure 1) and uterus (Figure 2A, black arrow, designated as PC3) were most likely derived from the two indicated green cells (Figures 2A–D insets), but this cannot be unambiguously determined using the preparation in Figure 2 alone.

**Figure 3:**
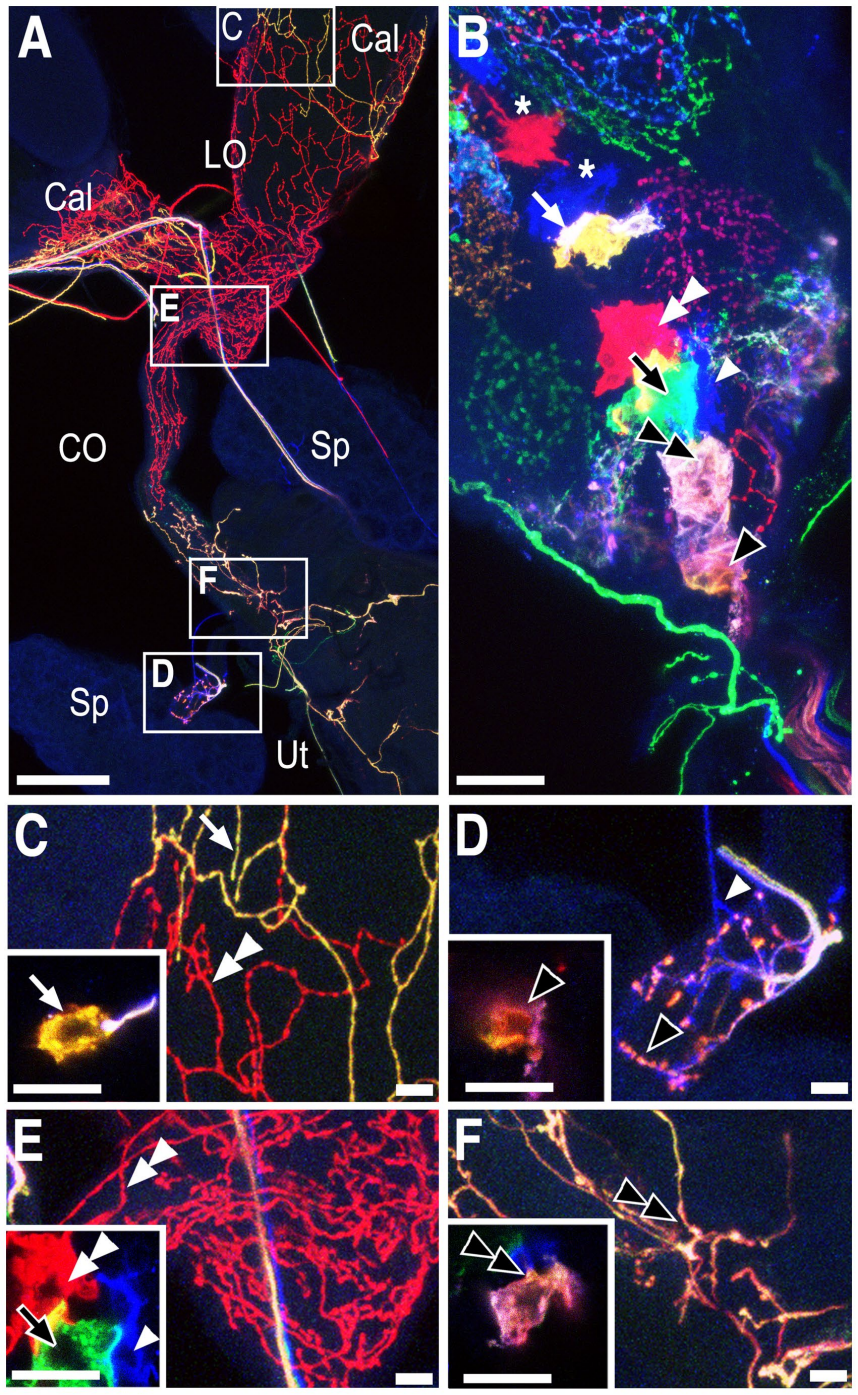
Neurons innervating the calyx, lateral oviducts, common oviduct, ovaries and spermathecae. Somata and/or processes from specific cells are indicated with matching white or black arrows/arrowheads in all panels. **(A)** Overview of the labeled reproductive tract with the spermathecae (Sp), calyx (Cal), lateral oviducts (LO), common oviduct (CO), and uterus (Ut) indicated. **(B)** A confocal stack of the VNC shows labeling of the cell bodies that project to either the reproductive tract (arrows and arrowheads) or the anterior neuropil (asterisks). **(C–F)** Higher magnification views of the boxed areas in panel **(A)** with insets showing optical slices of the cells indicated in panel **(B)**. Scale bars: **(A)**: 100 μm. All other scale bars: 10 μm.

Two cells, PC1 and PC2, innervate the posterior common oviduct and anterior uterus, one of which is labeled in the preparation shown in Figure 3 (Figures 3B,F, double black arrowhead). Both of these cells are labeled in Supplementary Figure S1. Also shown labeled in Figure 3 is a red cell body (Figures 3B,E inset, double white arrowheads) that broadly innervated both the lateral oviducts and the anterior common oviduct (Figures 3A,E, double white arrowheads) and is designated PC5. A more anterior red cell and a blue cell in this preparation projected into the anterior neuropil (Figure 3B, asterisks) rather than into the reproductive tract (data not shown). Additional labeled neurons in this preparation included SpA (Figures 3B,D, single black arrowhead, see also Supplementary Figure S1), SpB (Figures 3B–E inset, single white arrowhead, see also Figure 2) and a yellow cell (PC7, see Figure 1) that innervated the calyx (Figures 3B,C inset, white arrow). A large green cell (black arrows in Figure 3B and the 3E inset, PC3, see also Figure 2) innervated the posterior uterus in this preparation but the projections were relatively difficult to image (data not shown). Projections to the posterior uterus by PC3 could be more easily seen in other preparations (see Supplementary Figure S3).

**Figure 4:**
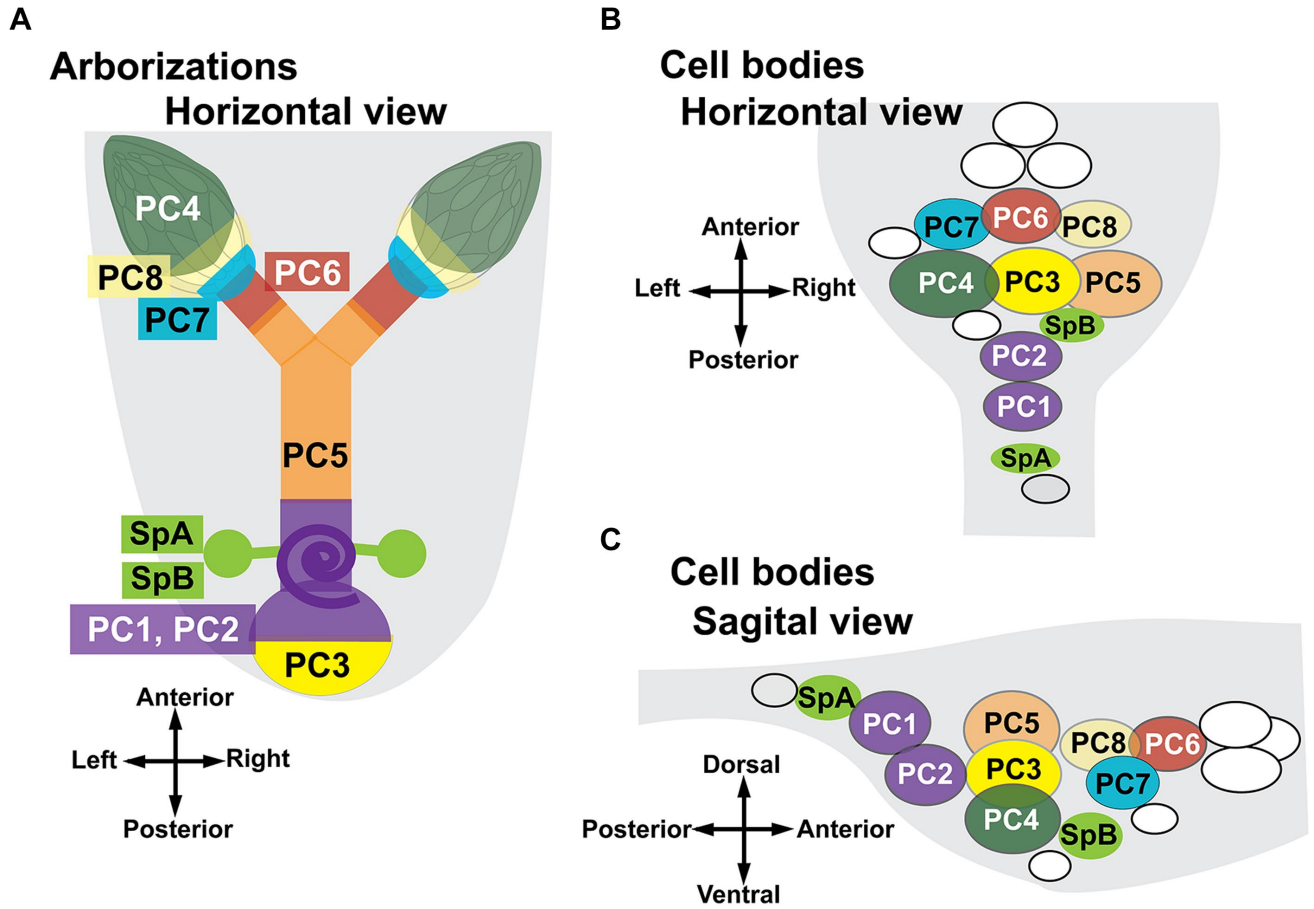
Summary of projections to the reproductive tract by identified cell bodies. The cartoons show a stylized horizontal view of the reproductive tract **(A)** and both horizontal **(B)** and sagittal **(C)** views of the ventral nerve cord. The purple spiral **(A)** represents the seminal receptacle. The parovarian glands are not shown. Regions of the reproductive tract are color-matched to the neuron(s) that innervate them. A total of 58 MCFO preparations were analyzed. The number of observations for specific patterns of innervation are listed here in parentheses. Two neurons innervate the spermathecae, SpA (5) and SpB (10). Eight numbered cells innervate other regions of the reproductive tract. PC 1+ 2: posterior common oviducts uterus and seminal receptacle (6); PC3: the posterior uterus (5); PC4: the ovaries (6); PC5: both lateral oviducts and the common oviduct (4); PC6: the calyx plus the lateral oviducts (4); PC7: the calyx alone (4); and PC8: the calyx plus additional processes that project into the base of the ovaries (3). The cells colored white in panels **(B,C)** are Tdc2(+) but do not project to the reproductive tract. The gray cell **(B,C)** may project to the parovarian glands but was clearly mapped in only one preparation.

A cartoon summarizing the MCFO data is shown in Figure 4. The targets in the reproductive tract (Figure 4A) are color-matched to the cells in the AbG (Figures 4B,C). Two cells innervate the stalks of the spermathecae and because they appeared smaller than other midline cells we have labeled them separately as SpA and SpB. Two cells that we have designated PC1 and PC2 project to the posterior common oviduct and the uterus and are anterior to SpA in the abdominal ganglion. A subcluster of at least three three large cells (PC3,4,5) is anterior to PC1 and 2 and includes cells that project to the uterus (PC3), the ovaries (PC4), and both the lateral and common oviducts (PC5). A group of three cells at the anterior end of the cluster innervates both the lateral oviducts and the calyx (PC6) the calyx alone (PC7) or both the calyx and the base of the ovaries (PC8). We detected at least two to three additional cells just anterior to PC6,7,8 that project anteriorly into the nerve cord rather than the reproductive tract and are colored white in Figure 4. Additional, more anterior Tdc2(+) cells are not shown. At least two small, nearby cell bodies are Tdc2(+) but did not appear to project to the reproductive tract and are also colored white in the cartoon. We detected one additional cell near SpA that appeared to project to the paraovarian glands. It was infrequently labeled and unambiguously mapped in only one preparation. We therefore we did not assign a specific designation to this cell and it is colored gray in Figure 4.

### A driver for a specific subset of neurons that innervate the calyx and lateral oviducts

**Figure 5:**
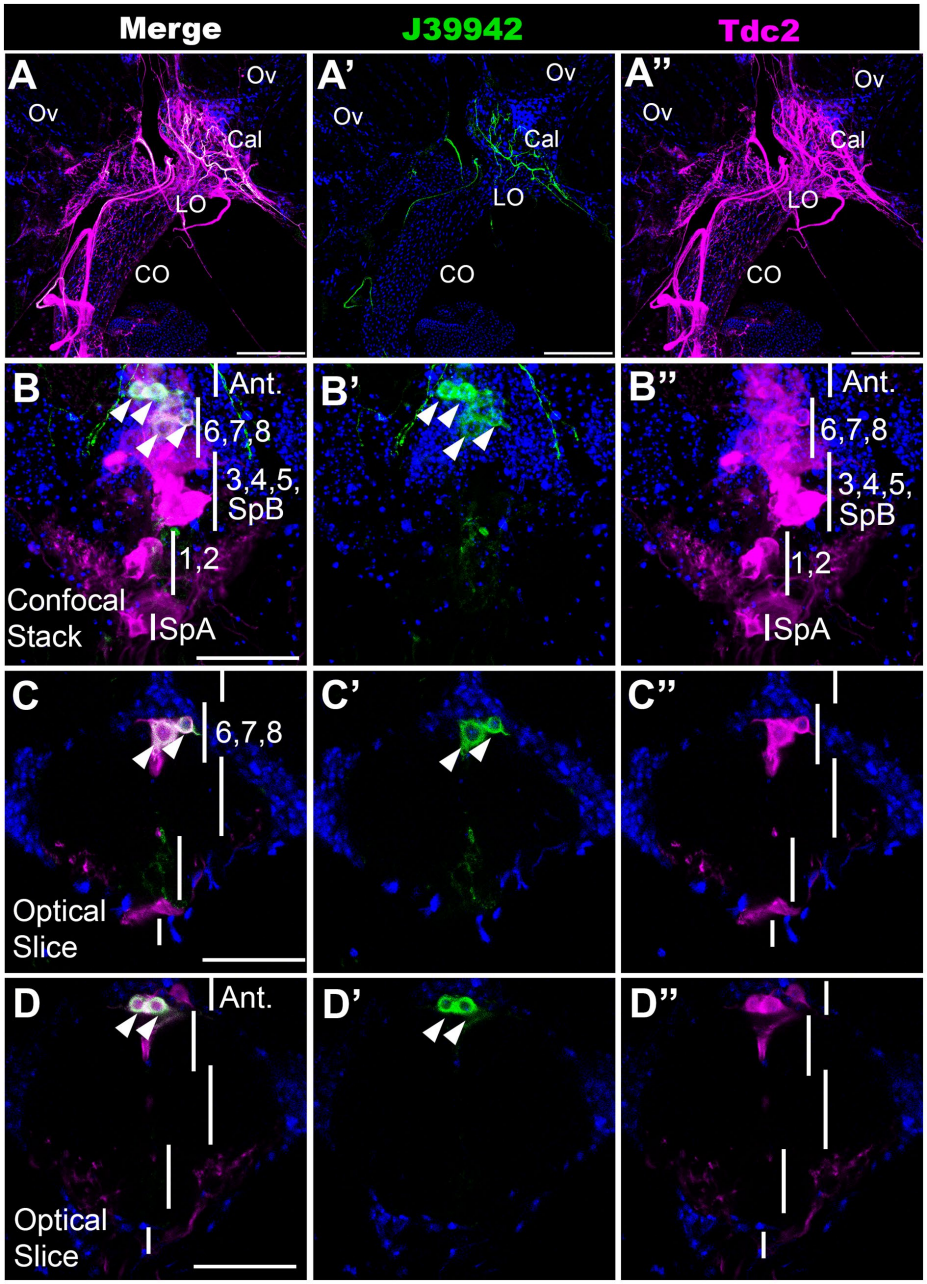
A subset of neurons that innervate the calyx. *J39942-Gal4* and *Tdc2-LexA* were used to express *UAS::mCD8-GFP* (green) and *LexAop::CD2-RFP* (magenta) respectively followed by the appropriate secondary antibodies. (Red was converted to magenta in Image J). **(A)** Confocal stack of the reproductive tract (horizontal view, 80 μm *z* projection, maximum signal) shows co-labeling in the calyx by processes expressing both *J39942-Gal4* and *Tdc2-LexA*. The ovaries (Ov), calyx (Cal), lateral oviducts (LO), and common oviduct (CO) are indicated. **(B–D)** A confocal stack **(B,B’,B’’)** and single optical slices of the VNC **(C,D,C’,D’,C’’,D’’)** show that *J39942-Gal4* labels four Tdc2(+) cells. Vertical white lines are regions that contain the indicated cells including SpA, PC1 and PC2, PC3-5 plus SpB, PC6-8 and cells that project to the anterior neuropil (“Ant.”). White arrowheads **(B,C,D)** indicate cells labeled with *J39942-Gal4*. Scale bars: **(A)** 100 μm. **(B–D)** 50 μm.

To further validate our MCFO mapping and begin to examine the function of specific subsets of neurons, we scanned a set of Gal4 drivers that employ regulatory regions of the *tyramine β hydroxylase* gene (Jenett et al., 2012; Meissner et al., 2023). We have previously shown that octopaminergic projections to the reproductive tract can optogenetically induce lateral oviduct contractions (Deshpande et al., 2022), and we therefore focused on one line associated with *tyramine β hydroxylase* (*J39942-Gal4 aka GMR76H07-Gal4*) that innervated this region (Figure 5A). To compare the expression pattern of *J39942-Gal4* with *Tdc2-LexA*, we co-expressed both drivers with the complementary markers *LexAop::CD2-RFP* and *UAS::mCD8-GFP*. We detected co-localization of *Tdc2-LexA* and *J39942-Gal4* in four cells at the anterior end of the posterior cluster (Figures 5B–D). We did not detect any additional *J39942-Gal4(+)* cells in this region that did not express *Tdc2-LexA*. Comparison of these images and additional co-labeling experiments (data not shown) to the data shown in Figures 1–3 suggest that that the two most anterior cells project to the anterior neuropil rather than the reproductive tract. Based on their location within the cluster and their arborization pattern, the two posterior cells labeled by *J39942-Gal4* that project to the reproductive tract are PC6 and 7.

### Optogenetic stimulation of a subset of octopaminergic neurons

**Figure 6:**
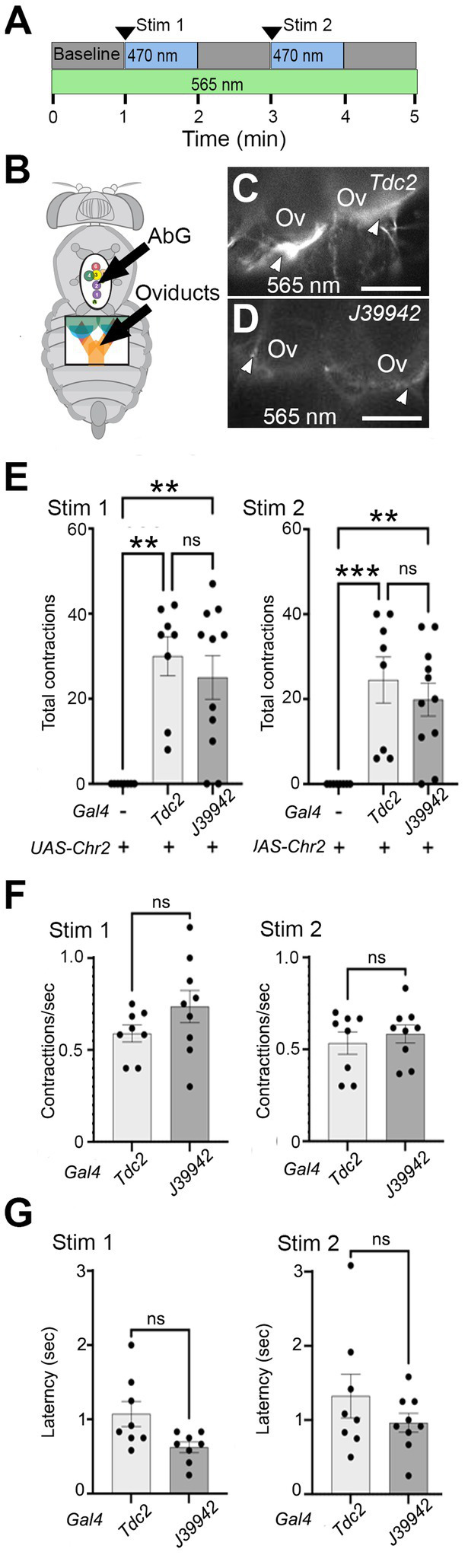
Stimulating a subset of neurons initiates lateral oviduct contraction. **(A)** The optogenetic protocol included a baseline followed by two 1 min periods of stimulation with a ~ 470 nm LED and intervening 1 min periods without stimulation. The oviducts were visualized with a ~ 565 nm LED. **(B)** Small windows cut in the ventral cuticle of the thorax (black oval) and abdomen (black rectangle) allowed stimulation of cells in the AbG and visualization of the oviducts, respectively. **(C,D)** Processes at the base of the ovaries (Ov) that express *UAS-ChR2XXM-TdTomato* (white arrowheads) with either *Tdc2-Gal4* **(C)** or *J39942-Gal4* **(D)** were visualized with ~565 nm excitation. **(E–G)** Total number of contractions seen in each stimulation period **(E)**, the rate of contractions **(F)**, and average latency to the contractions **(G)** are indicated for each genotype (*n* = 8 for control without Gal4 and for *Tdc2-Gal4*, *n* = 11 for *J39942-Gal4*). A Kruskal-Wallis test was used for the analysis in panel **(E)** (*p* = 0.0008 and 0.0004 for Stim 1 and Stim 2, respectively) with multiple comparisons ^**^p = 0.0015–0.006; ^***^p = 0.0008. **(F,G)** Mann–Whitney tests of frequency and latency respectively; ns, not statistically significant by Mann–Whitney. Scale Bars: 100 μm.

Projection to the calyx of the cells labeled by line *J39942-Gal4* predicted that they could potentially play a role in regulating the function of this region. Alternatively, it remained possible that all cells in the posterior cluster might be required for lateral oviduct contractions and perhaps other functions previously assigned to octopaminergic signaling pathways. To distinguish between these possibilities, we compared the effects of optogenetically stimulating all Tdc2(+) (neurons) vs. the subset labeled by *J39942-Gal4* (Figures 6A,B). We used the channelrhodopsin variant *ChR2-XXM* which is directly conjugated to TdTomato (Scholz et al., 2017), thus allowing visualization of projections labeled with either *Tdc2-* (Figure 6C) or *J39942-Gal4* (Figure 6D). The difference in the intensity of the fluorescent signals appear to be consistent with the expression of *J39942-Gal4* in a subset of the Tdc2(+) that innervate the calyx. However, differences between the expression of *ChR2-XXM* in the cells co-labeled by both drivers are also possible.

We found that stimulating either the entire Tdc2(+) posterior cluster or the subset labeled by *J39942-Gal4* in these preparations was followed by repetitive contractions that were similar in number (Figure 6E) and frequency (Figure 6F); the latency between optogenetic stimulation and the onset of contractions appeared slightly shorter for *J39942-Gal4* than *Tdc2-Gal4* but this was not statistically significant (Figure 6G). These data support the idea that specific subsets of cells within the posterior cluster rather than the cluster as a whole may be sufficient to mediate at least one of the functions proposed for octopaminergic signaling in the reproductive tract.

### Two neurons in the posterior cluster are differentially excitable

**Figure 7:**
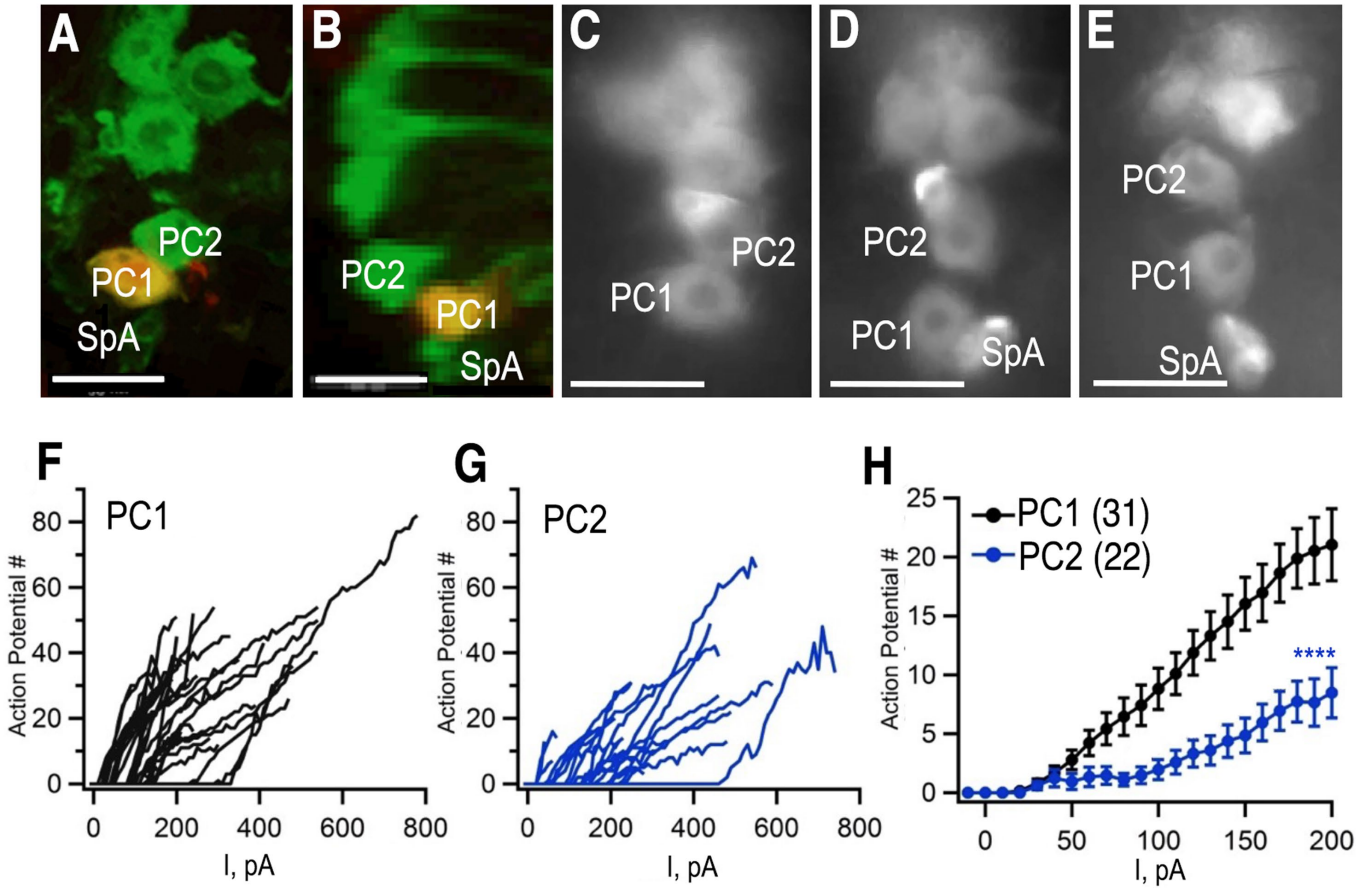
Whole cell patch clamp to measure excitability. **(A,B)** *Tdc2-Gal4* was used to express *UAS-mCD8-GFP* and labeled with anti-GFP (green). PC1 was injected with biocytin (yellow) in this preparation. **(A)** Confocal image of PC1 and PC2 with the VNC in a horizontal orientation. **(B)** The same confocal stack shown in panel **(A)** was digitally rotated ~90 degrees. **(C–E)** Three additional examples of preparations used for recording showing variations in the distance between PC1 and PC2 and the variable presence of SpA in the field of view. **(F,G)** The number of action potentials vs. current injection of PC1 **(F)** and PC2 **(G)** measured in whole-cell current clamp mode. **(H)** Average of PC1 (*n* = 31) and PC2 (*n* = 22, mean + SEM). Regression analysis (see the section Materials and methods) with ^****^p< 2 × 10^−16^ and a least squares estimate of 5.72 action potentials. Scale Bars: 10 μm. The average resting potentials of PC1 and PC1 were − 52.5 ± 7.3 mV (mean ± standard deviation; median: − 53.2 mV; *n* = 35) and − 51.2±−4.7 mV (median: − 51.2 mV; *n* = 22) respectively and neither mean (Student’s *t* test) nor median (Mann Whitney test) were significantly different.

To complement our studies on neuroanatomical diversity, we performed additional electrophysiological experiments. Previous electrophysiological studies of octopaminergic neurons have been performed in larger insects including the locust, as well as crustaceans such as the lobster (Duch et al., 1999; Grolleau and Lapied, 2000; Heinrich et al., 2000; Heidel and Pfluger, 2006). To probe the electrophysiological properties of octopaminergic neurons in *Drosophila*, we performed whole cell, patch clamp recordings from Tdc2(+) cell bodies in the abdominal ganglion using previously described methods (Harrigan et al., 2020). To label the Tdc2(+) cells, we expressed the marker mCD8-GFP using *Tdc2-Gal4*, the same driver we used for MCFO experiments. The GFP marker was easily visualized after fixation (Figures 7A,B) and in live images while patching (Figures 7C–E). We chose to focus on the two large cell bodies at the posterior tip of the cluster because they could be easily visualized and consistently distinguished from each other and the rest of the cluster (e.g., in the three preparations shown in Figures 7C–E). These cells correspond to PC1 and PC2 in Figures 1–4.

To further confirm that we were recording from the same cells that we had imaged using MCFO, we injected biocytin into cells during a subset of recordings (Figures 7A,B). Images of a horizontally oriented ventral nerve cord (Figure 7A), and digital rotation (Figure 7B) following injection into PC1 confirm that is dorsal and posterior to PC2. Access to ventral nerve cord for electrophysiological recordings required disruption of the glial sheath that surrounds it, which led to slight changes in the absolute position of the octopaminergic neurons when visualized after fixation (Figures 7A,B) or during the patch clamp experiments (Figures 7C–E). PC1 and 2 could nonetheless be consistently identified as the first and second large, midline cells at the posterior tip of the cluster (Figures 7A–E).

Using whole cell path clamp in current clamp, we detected relatively few spontaneous action potentials in either PC1 or PC2 in our initial, baseline recordings (data not shown). Similarly, octopaminergic neurons are generally silent at baseline in the lobster ventral nerve cord (Heinrich et al., 2000). To determine if a baseline inhibitory potential was responsible for the apparent quiescence of the cells, step current pulses of increasing amplitude were injected and the number of action potentials after each injection was recorded (Figures 7F,G). The number of action potentials elicited by each current step was significantly higher for PC1 (Figures 7F,H) compared to PC2 (Figures 7G,H). These data suggest that PC2 may be inherently less excitable than PC1 or receive stronger inhibitory inputs.

**Figure 8:**
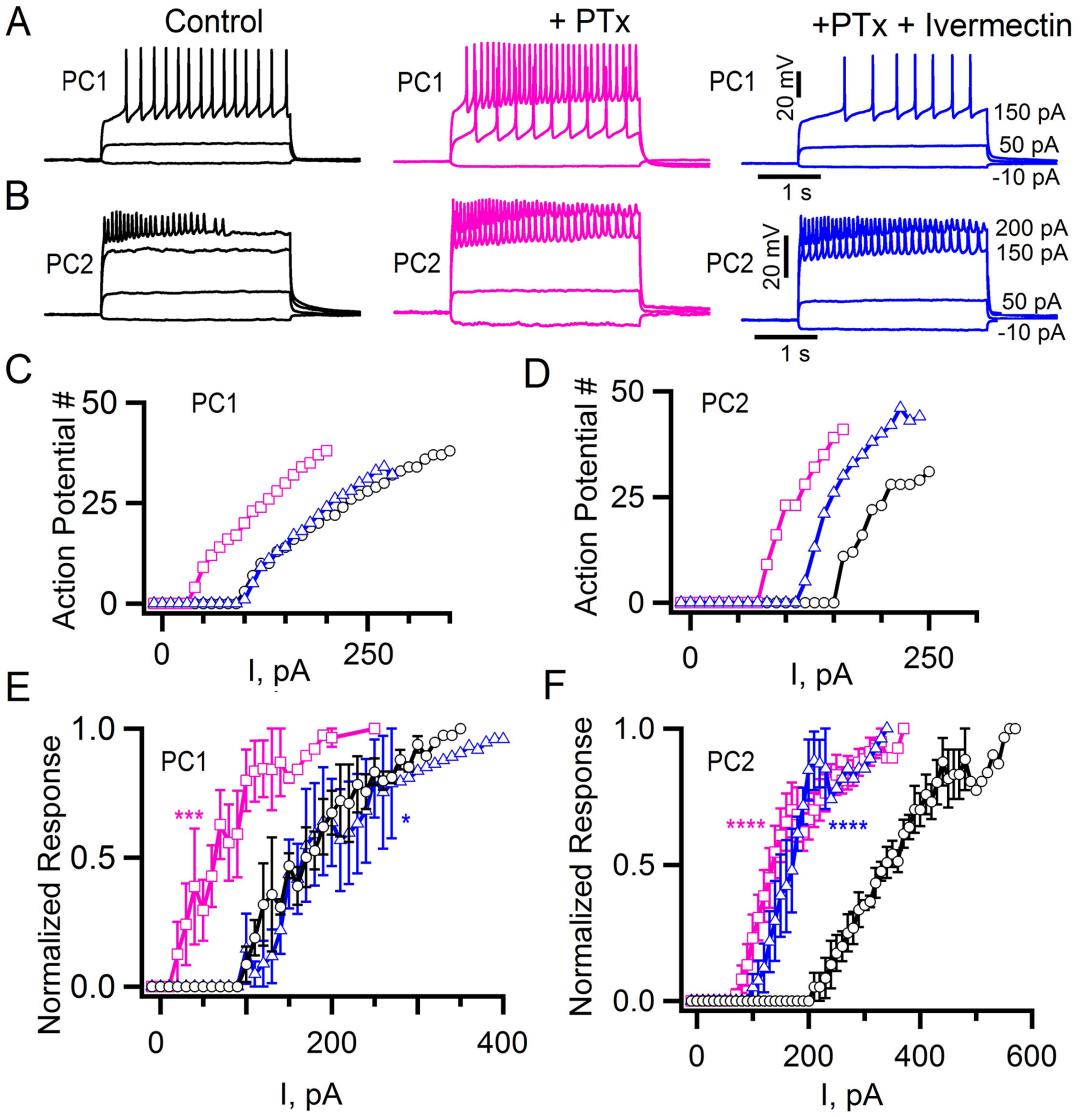
Picrotoxin and ivermectin effects on tonic inhibition. **(A,B)** Action potentials elicited at the indicated current injections in control cells, after application of 100 μM Picrotoxin, (PTx, pink) and in the presence of 100 μM PTx + 1 μM Ivermectin (blue) for PC1 **(A)** and PC2 **(B)**. **(C,D)** The number of action potentials vs. current injections for the PC1 **(C)** and PC2 **(D)** cells shown in panels **(A,B)**, respectively, and treated with PTx alone (pink squares), PTx + Ivermectin (blue triangles) or saline alone control (black circles). **(E,F)** The mean normalized response for PC1 (**E**: control, *n* = 5; +PTx, *n* = 5; +PTx + Ivermectin, *n* = 4) and PC2 (**F**: control, *n* = 6; +PTx = 6; +PTx + Ivermectin, *n* = 3). Regression analysis (see Methods) with ^****^p < 2 × 10^−16^, ^***^p = 2.67 × 10^−11^ and ^*^p = 0.029 for PTx or PTx + Ivermectin compared to control. The least squares estimate of the coefficient of picrotoxin for PC1 and PC2 were 0.296 and 0.316 and for ivermectin −0.089 and 0.262, respectively, with negative vs. positive values indicating shifts in opposite directions.

To explore whether differences in excitability are due to different levels of tonic, inhibition in PC1 and PC2 neurons, we bath applied the GABA Cl^−^ channel blocker picrotoxin (Ffrench-Constant et al., 1991, 1993; Stilwell et al., 2006). We again injected current in a stepwise fashion and quantified the number of action potentials that were elicited, both before and after treatment with picrotoxin (Figures 8A,B). Application of 100 μM picrotoxin resulted in a significant change of the current-response curve in both PC1 and PC2 neurons (Figures 8C–F, magenta squares). The mean current required to elicit at least 10% of the maximum number of action potentials substantially decreased in both cell types (PC1 control: 153 ± 42 pA; PC1 picrotoxin 43 ± 7 pA; PC2 control: 248 ± 53 pA PC2 picrotoxin: 100 ± 7). This indicates that tonic inhibition contributes to a reduced excitability in both cell types and that PC2 cells are intrinsically less excitable.

In addition to GABA gated inhibitory channels, *Drosophila* express a glutamate-gated chloride channel (GluCl) that is also responsive to picrotoxin (Cully et al., 1996; Etter et al., 1999). We are not aware of a specific GluCl antagonist. Therefore, to determine whether GluCl might contribute to the inhibitory control of PC1 and/or PC2, we tested the effects of the GluCl agonist ivermectin (Cully et al., 1996; Kane et al., 2000). Since both PC1 and PC2 were relatively quiescent at baseline, we tested the effects of ivermectin after first applying picrotoxin. We detected a shift in the current-response curves of both PC1 and PC2 in response to ivermectin following picrotoxin (Figures 8C–F, blue triangles). Activation of GluCl appeared to more effectively restore the level of inhibition seen prior to the initial application of picrotoxin for PC1 compared to PC2 (Figures 8C–F, blue triangles). These data further underscore the subtle differences between these two cells and suggest that GluCl may play a relatively more important role in the baseline inhibition of PC1 compared to PC2.

### Expression of the GluCl receptor

**Figure 9:**
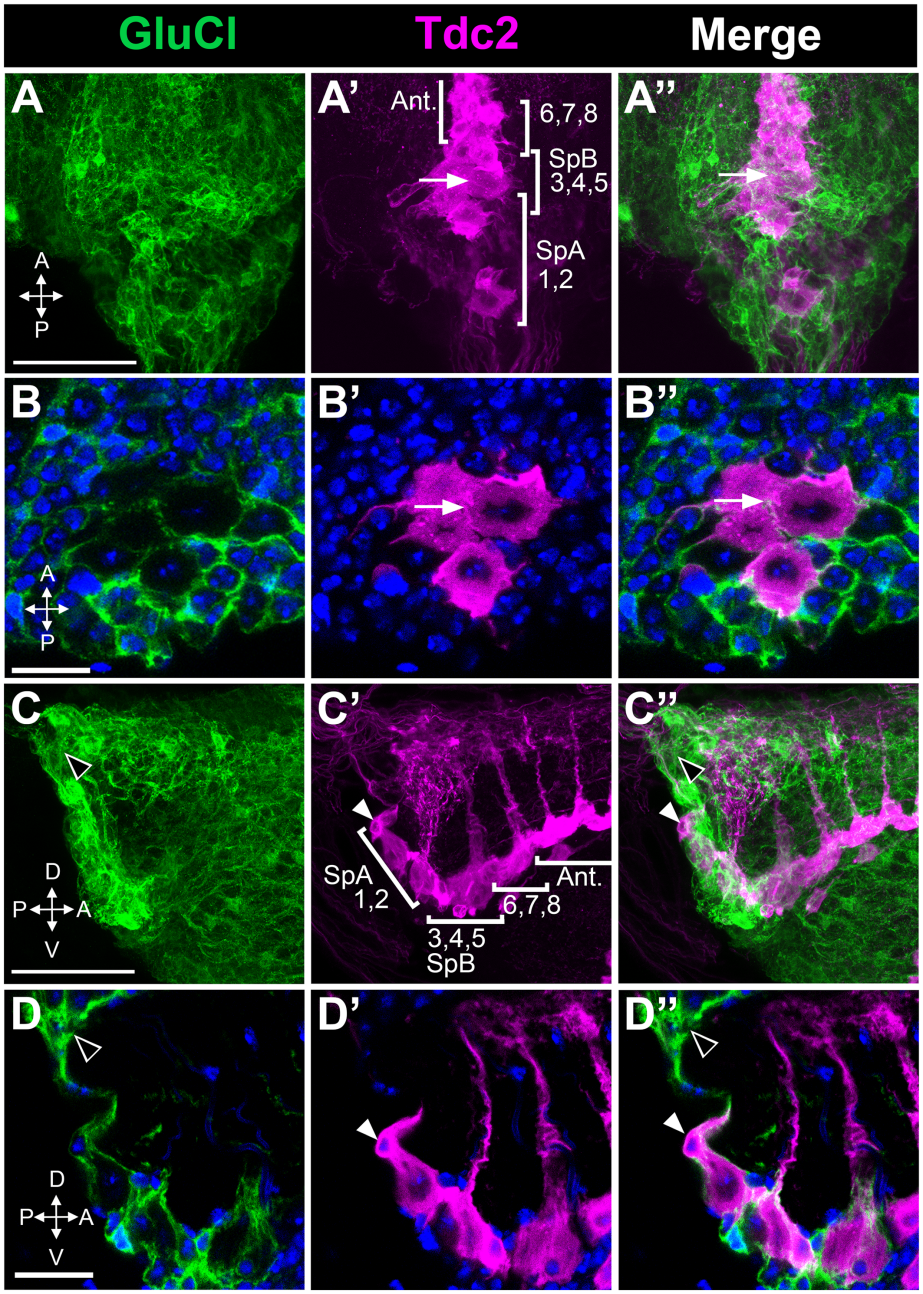
Octopaminergic neurons in the posterior cluster do not express the GluCl receptor. *GluCl-Gal4* expression was compared to *Tdc2-LexA* expression using the reporters *UAS::mCD8-GFP* and *LexAop::CD2-RFP*. **(A,A’,A’’)** Dorsal to ventral maximum signal projection through the abdominal ganglion with the neurons indicated as in Figure 5. **(B,B’,B’’)** Single slice images comparing epitope expression with one of the neurons in the PC3-5 subgroup indicated (white arrow). **(C,C’,C’’)** Sagittal view, maximum signal projection through the abdominal ganglion with the neurons indicated as in Figure 5. White and black arrowheads indicate the SpA cell and GluCl(+) labeling, respectively. **(D,D’,D’’)** Single optical slice from the sagittal stack (arrowheads as in **C**). Scale Bars: **(A,C)**: 50 μm. **(B)**: 5 μm. **(D)**: 10 μm.

Inhibitory receptors expressed in PC1 and PC2 could potentially be responsible for the effects of picrotoxin and ivermectin that we observed. Alternatively, these effects could be mediated by inhibitory receptors expressed on other neurons that innervate PC1 and PC2. While several GABA subunits are expressed in *Drosophila* there is only one GluCl gene, thereby simplifying the analysis of GluCl expression (Cully et al., 1996; Etter et al., 1999; Liu and Wilson, 2013). To determine the expression pattern of GluCl, we used the MiMIC line *GluCl-MiMIC-Gal4* (Lee et al., 2018). We co-labeled tissue using *GluCl-MiMIC-Gal4* and *Tdc2-LexA* to express the green and red markers mCD8-GFP and CD2-RFP, respectively. We detect extensive labeling of processes near both the Tdc2(+) somata that are GluCl(+) (Figure 9). However, it is possible that Tdc2(+) cell bodies also show low levels of *GluCl-Gal4* expression (Figure 9). These data suggest that the effects of ivermectin on octopaminergic cells in this cluster may be mediated directly or indirectly by GluCl expressed on Tdc2(+) or other cells, and further experiments will be needed to differentiate between these two possibilities.

## Discussion

The Tdc2(+) cluster in the abdominal ganglion that innervates the reproductive tract provides a useful model to determine how individual neurons within an aminergic cluster may regulate distal targets, analogous to the projections from aminergic nuclei in mammalian brain. However, in contrast to mammalian nuclei, the cluster that innervates the reproductive tract contains a small number of cells, thus simplifying its analysis. The stereotyped position of invertebrate neurons also facilitates electrophysiological studies, similar to those pioneered in crustaceans and larger insects (Goodman and Spitzer, 1981; Harris-Warrick and Marder, 1991; Heinrich et al., 2000).

A cluster of Tdc2(+) cells at the posterior end of the abdominal ganglion as well as their combined projections into the reproductive tract has been previously identified (Monastirioti, 2003; Rodriguez-Valentin et al., 2006; Rezaval et al., 2014; Pauls et al., 2018; White et al., 2021). Assessment of the number of octopaminergic cells in this region has varied; the low end of the range (5–6) is likely to be an underestimate based on the relatively low sensitivity of the method used to labels the cells (Monastirioti et al., 1995; Monastirioti, 1999). A higher estimate appears to include octopaminergic neurons that are anterior to those that project to the reproductive tract (Schneider et al., 2012), and we have designated those that project to the reproductive tract as the posterior cluster within the abdominal ganglion. We speculate that the posterior cluster is essentially equivalent to the group of 8–9 octopaminergic neurons that co-express *Tdc2* and the sex-specific gene *doublesex* (Rezaval et al., 2014). These cells are present in females but not in males and innervate the reproductive tract (Rezaval et al., 2014). Silencing *Tdc2/dsx* cells changes a variety of post-mating behaviors including egg-laying (Rezaval et al., 2014).

It was possible that all of the *Tdc2/dsx* cells (or other octopaminergic neurons) could project diffusely throughout the reproductive tract, perhaps regulating downstream targets as a group. Conversely, we find that each cell in this region that we have mapped innervates relatively distinct but overlapping targets. These include two cells each that innervate the spermatheca and posterior common oviduct, and three that innervate the calyx ± portions of the ovaries or lateral oviduct. We detect one cell each for innervation of the ovaries, the posterior uterus and a region that includes both the lateral and common oviducts.

In mammals, aminergic nuclei have historically been treated as relatively homogenous structures that mediate aminergic “tone.” RNA seq studies show that neurons within the raphe are transcriptionally diverse and functional studies of both the locus coeruleus and raphe have revealed subpopulations that have unexpectedly distinct effects on behavior (Soiza-Reilly and Commons, 2014; Chandler et al., 2019; Huang et al., 2019; Borodovitsyna et al., 2020; Okaty et al., 2020; Poe et al., 2020). Our data similarly show that a small cluster of aminergic of cells can nonetheless have divergent targets. These data are also consistent with studies in locust in which subpopulations of octopaminergic neurons mediate distinct effects (Duch et al., 1999).

The fly connectome has been previously mapped using electron microscopy and 3D reconstruction using serial sections (Scheffer et al., 2020; Dorkenwald et al., 2023; Schlegel et al., 2023; Winding et al., 2023). The length of the processes that project from the ventral nerve cord to the reproductive tract render a similar reconstruction technically difficult. The use of non-synaptic modes of neuronal communication by many aminergic neurons also preclude molecular techniques that require close synaptic contacts (Feinberg et al., 2008; Talay et al., 2017; Shearin et al., 2018). Some octopaminergic neurons in the central brain are likely to signal via true synaptic connections (Wasserman et al., 2015; Wong et al., 2021). However, using trans-Tango (Talay et al., 2017) with *Tdc2-Gal4* as a presynaptic partner we were unable to detect post-synaptic labeling of any targets in the reproductive tract (data not shown). These data indicate that few, if any of the octopaminergic/tyraminergic projections to the reproductive signal via true synaptic transmission. Rather, signaling at these sites is likely to occur via volume transmission and the release of octopamine and or tyramine from large dense core vesicles (Hoyle et al., 1980; Watson and Schurmann, 2002; Fuxe et al., 2010; Stocker et al., 2018). In the absence of true synaptic connections for octopaminergic projections into the reproductive tract, the methods used previously to map the fly connectome in the central nervous system are not feasible, highlighting the importance of the current data for understanding the neuroanatomy of this region.

Three cells within the cluster that we have studied have relatively distinct anatomic targets (PC3, 4, and 5), consistent with the possibility that they may mediate diverse functions in the uterus, the ovaries and the oviducts, respectively. By contrast, the other cells in the cluster appear to have overlapping projection patterns. These include the two cells that appear to project to similar sites in the distal portion of the posterior oviduct and anterior uterus (PC1 and 2), two that innervate the stalk of the spermatheca (SpA and B) and three cells that show overlapping patterns that include the calyx (PC6, 7 and 8).

It is possible that one or both of SpA and/or B are responsible for the octopaminergic and/or tyraminergic regulation of the spermatheca (Avila et al., 2012). Similarly, it is possible that any one of the four cells that either send processes to the ovary as a whole (PC4), or the calyx where eggs exit the ovary (PC6, 7, 8) could play a role in ovulation (Deady and Sun, 2015; Meiselman et al., 2018). PC5, 6, 7 or 8 could potentially contribute to regulation of lateral oviduct contractility (Rodriguez-Valentin et al., 2006; Deshpande et al., 2022). While the base of the ovaries is innervated by both PC4 and PC8, the more anterior regions of the ovaries are only innervated by PC4. Therefore, contractions of the peritoneal sheath in the more anterior regions would most likely be regulated by PC4 rather than PC8 (Middleton et al., 2006; Meiselman et al., 2018). Similarly, only the projections of PC4 would be able to influence any developmental effects linked to octopamine that may occur in the anterior regions of the ovaries (Andreatta et al., 2018; Meiselman et al., 2018; Yoshinari et al., 2020; Kim et al., 2021).

We have previously shown that optogenetic activation of all Tdc2(+) neurons can initiate contractions in the lateral oviducts and calyxes (Deshpande et al., 2022). Our data using a more restrictive driver (*J39942-Gal4* aka *GMR76H07-Gal4*) indicate that a subset of Tdc2(+) cells can have similar, if not identical effects. Additional experiments will be needed to determine if all the cells that innervate these regions have the same effect. It is possible that the different octopaminergic cells that innervate the calyx and lateral oviducts represent alternative, and essentially redundant pathways to elicit the same response. As suggested for some pathways within the stomatogastric ganglion of the crab, this may be essential to ensure a robust response under a variety of conditions (Gorur-Shandilya et al., 2022). Alternatively, it is also possible that each of these cells could serve a distinct function to induce oviduct contractions under different contexts or in coordination with a different subset of neurons. Potential partners include glutamatergic/ILP7(+) cells which innervate the reproductive tract and can induce oviduct contractions (Castellanos et al., 2013; Gou et al., 2014; Deshpande et al., 2022). Additional drivers for subsets of other octopaminergic neurons will be needed to address these questions.

The sites in the reproductive tract that we have anatomically mapped to specific neurons in the nerve cord may be directly regulated by local octopamine release. However, we cannot rule out a contribution of neurohumeral release from the CNS (Braunig, 1995). It is also possible that other less direct octopaminergic pathways play an important role in regulating octopamine-dependent activities within the reproductive tract. In both insects and crustaceans, octopamine can alter the morphology and activity of presynaptic axons and nerve terminals and thereby regulate the response of downstream targets via indirect mechanisms (Breen and Atwood, 1983; Nishikawa and Kidokoro, 1999; Goaillard et al., 2004; Koon and Budnik, 2012). In the reproductive tract, we have shown that presynaptic glutamatergic nerve terminals express *Octβ2R* (Deshpande et al., 2022) and these cells regulate contractility of the oviduct (Castellanos et al., 2013; Gou et al., 2014). Octopaminergic projections may also exert indirect control of the reproductive tract via octopamine receptors that are expressed on peripheral interneurons. At least 26 cells expressing the channel ppk1 are expressed in the reproductive tract and a subset have been proposed to function as mechanosensory cells to regulate the activity of glutamatergic projections from the abdominal ganglion (Yang et al., 2009; Rezaval et al., 2012; Gou et al., 2014; Lee et al., 2016; Wang et al., 2020a). We have previously reported that many, if not all of the neurons in this subset also express one or more subtypes of the six of the known octopamine receptors (Deshpande et al., 2022; Rohrbach et al., 2024). It is therefore possible that octopaminergic receptor activation in peripheral ppk1 neurons could signal to other sites within the reproductive tract that are distal from the octopaminergic projections we have mapped.

Additional indirect pathways may be mediated by octopamine receptors expressed on non-neuronal tissue. We are unable to detect octopamine receptors on any muscle cells within the reproductive tract (Deshpande et al., 2022, Rohrbach et al., 2024). However, genetic rescue experiments indicate that the octopamine receptors expressed in epithelial cells that line the oviduct are required for egg-laying, and the epithelium may signal to adjacent muscle tissue (Lee et al., 2003, 2009; Lim et al., 2014). Together, these observations raise the possibility that a complex web of regulatory interactions may exist beyond the direct octopaminergic projections that we have mapped.

The stereotyped position of invertebrate neurons facilitates electrophysiological studies, e.g., those pioneered in crustaceans and larger insects (Goodman and Spitzer, 1981; Harris-Warrick and Marder, 1991; Heinrich et al., 2000). We have exploited this property to compare two nearby cells, PC1 and 2. Although the size and location of their cell bodies and their projection patterns are similar, their intrinsic excitability and their response to the GluCl receptor agonist ivermectin differs. Differences in the electrophysiological properties of similar, unpaired medial neurons have also been identified in larger insects, and some of the channels that might be responsible for these differences have been characterized (Goodman and Spitzer, 1981; Grolleau and Lapied, 2000; Heidel and Pfluger, 2006). Future electrophysiological experiments with other neurons in the posterior cluster will provide an important comparison to PC1 and PC2; however, movement of the cells following disruption of the glial sheath makes it difficult to unambiguously identify most of these cells when they are all labeled with *Tdc2-Gal4*. The use of additional drivers that label subsets of the cells within the cluster such as *J39942-Gal4* will facilitate future electrophysiological experiments.

We find that activation of GluCl can increase excitation of PC1 and to a lesser extent PC2. Compared to the surrounding neuropil, GluCl appears to be expressed at relatively low levels in the Tdc2(+) neurons within the posterior cluster. These data suggest that the regulation of octopaminergic cells in the posterior cluster may include indirect inhibitory pathways. RNA seq studies may yield important clues about the identity of local interneurons that express GluCl and GABA receptors and could potentially innervate the posterior cluster (Allen et al., 2020; Li et al., 2022). In addition, further electrophysiological studies of octopaminergic neurons in *Drosophila* will be important to help define the mechanisms that determine the differences in excitability we have observed and their physiological role.

## Data availability statement

The original contributions presented in the study are included in the article/Supplementary material, further inquiries can be directed to the corresponding author.

## Ethics statement

The manuscript presents research on animals that do not require ethical approval for their study.

## Author contributions

ER: Conceptualization, Writing – original draft, Writing – review & editing, Formal analysis, Investigation. JA: Formal analysis, Investigation, Visualization, Writing – review & editing, Validation. PM: Formal analysis, Investigation, Visualization, Writing – review & editing, Conceptualization, Methodology. MK: Formal analysis, Investigation, Writing – review & editing. SD: Conceptualization, Writing – review & editing. FS: Formal analysis, Investigation, Writing – review & editing, Conceptualization, Methodology, Supervision. DK: Conceptualization, Supervision, Writing – review & editing, Funding acquisition, Project administration, Resources, Writing – original draft.

## Supplementary material

SUPPLEMENTARY FIGURE S1 A neuron at the tip of the posterior cluster projects to the spermathecae. **(A)** Overview of the labeled reproductive tract with the common oviduct (CO), spermatheca (Sp), Ovary and uterus (Ut) indicated. **(B)** Labeling of the abdominal ganglion shown as a confocal stack. The white arrow in panel **(B)** indicates the specific labeling of a yellow cell, allowing an unambiguous match to yellow processes in the reproductive tract. This cell is indicated as SpA in Figure 4. The white arrowhead and asterisk indicate two cells that are similarly labeled red and therefore cannot be assigned to processes in the reproductive tract based on this preparation alone. Based on other labelings, the larger more posterior cell is PC1 and the smaller, anterior cell is SpB. At least one large blue cell body is labeled (double white arrowheads), but additional, smaller blue cell bodes appear to be labeled blue in this preparation. Based on other labelings, the large blue cell is PC4, which innervates the ovaries. Neuropil anterior to the posterior cluster is labeled “Ant Npl”. Panels **(C,D,E)** correspond to boxed areas in panel **(A)** and include the stalk of the spermatheca **(C)**, the anterior uterus **(D)**, and the ovaries **(E)**. Insets in panels **(C–E)** represent single optical sections of the confocal stack shown in panel **(B)**. **(C)** Yellow processes correspond to the yellow cell (SpA) in both the inset and panel **(B)** (white arrows). On the basis of other labeling experiments, the red processes (asterisks) correspond to the small red cell labeled with an asterisk in panel B (SpB in Figure 4). **(D)** On the basis of other labelings, the red processes in the uterus and posterior oviduct are derived from the indicated red cell (white arrowhead in inset) and represent PC1. **(E)** The blue processes correspond to large blue cell innervating the ovaries (white double arrowheads in inset and in panel **B**) and is indicated as PC4 in Figure 4. Scale bars: **(A,C–E)**: 50 μm. **(B)** and insets in **(C,D,E)**: 10 μm.

SUPPLEMENTARY FIGURE S2 Two neurons innervate the posterior common oviduct and uterus. **(A)** Overview of the labeled reproductive tract with the ovaries (Ov), common oviduct, (CO), spermathecae (Sp), and uterus (Ut) indicated. Red processes in the ovaries (black arrowheads) and spermathecae (black arrows) are also indicated. **(B,C)** The boxed regions from panel **(A)** shown at higher magnification include the posterior common oviduct **(B)** and the uterus **(C)** with blue (white arrowhead), green (white arrow) and red (black arrow) processes indicated. **(D)** Labeled cells in the abdominal ganglion include one blue (white arrowhead) and one green (white arrow) cell represented as PC1 and PC2 in Figure 4, respectively. Two red cells are visible. Based on a comparison to other labelings, the smaller red cell (asterisk) and the larger red cell (black arrowhead) innervate the stalk of the spermathecae and the ovaries respectively and are indicated as SpB and PC4 in Figure 4. Scale bars: **(A)**: 50 μm. **(B–D)** and inset in **(D)**:10 μm.

SUPPLEMENTARY FIGURE S3 A neuron innervating the posterior uterus. **(A)** Overview of the labeled reproductive tract with the calyx (Cal), lateral oviduct (LO), common oviduct (CO) and uterus (Ut) indicated. The panel **(A)** inset shows labeling of the abdominal ganglion (AbG) as a confocal stack and includes one red cell (single white arrowhead), one blue cell (white arrow), and two green cells (asterisk and double white arrowhead). **(B–D)** Higher magnification of the boxed regions in panel **(A)**. Insets in panels **(B–D)** show single optical sections of cells indicated in the panel **(A)** inset. Identification of the red cell as PC1 (projecting to the posterior oviduct and anterior uterus) and the blue cell as PC6 (projecting to the lateral oviduct and calyx) can be determined from this preparation alone since only one red cell and one blue cell were observed in the abdominal ganglion. Comparison of this preparation to other labelings allows assignment of the small green cell (asterisk) as PC7 (projecting to the calyx), and the larger green cell as PC3 (double white arrowheads, projecting to the posterior uterus). Scale bars: **(A)**: 100 μm. **(B–D)** and insets in **(A–D)**: 10 μm.
