# Supplementary figures and images for: Heterogeneity in the projections and excitability of tyraminergic/octopaminergic neurons that innervate the Drosophila reproductive tract
Source: Front Mol Neurosci. 2024 Aug 2;17:1374896. doi: 10.3389/fnmol.2024.1374896 (PMC11327148; doi:10.3389/fnmol.2024.1374896)

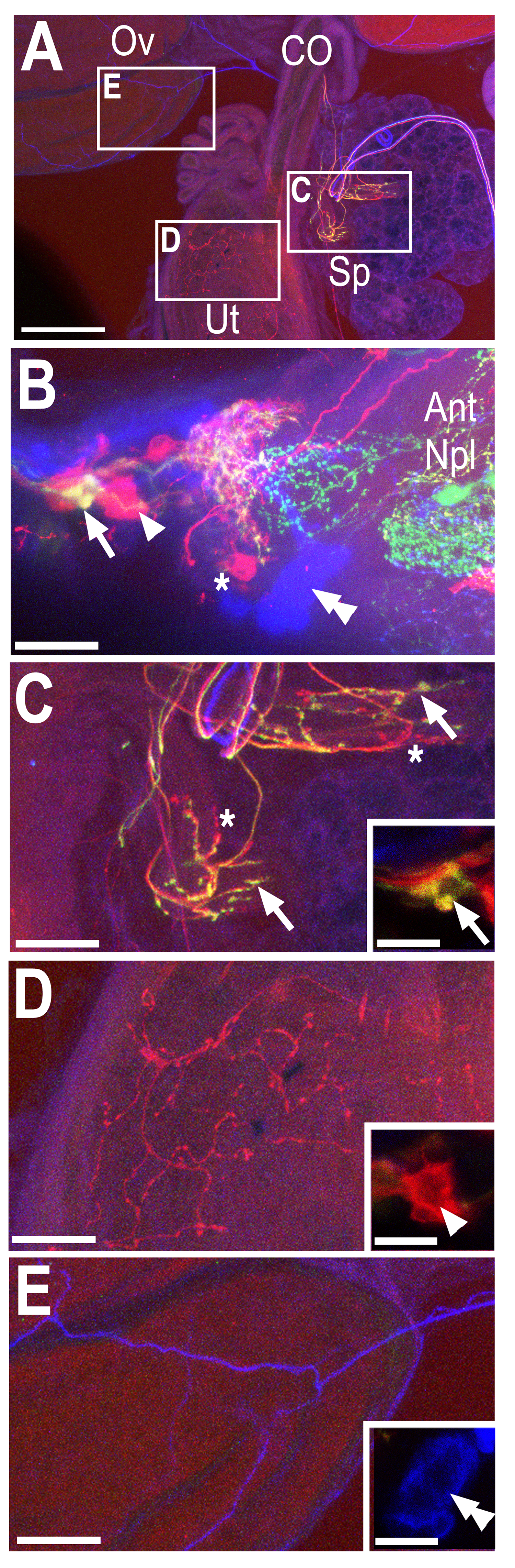

Supplement: SUPPLEMENTARY FIGURE S1 — A neuron at the tip of the posterior cluster projects to the spermathecae. (A) Overview of the labeled reproductive tract with the common oviduct (CO), spermatheca (Sp), Ovary and uterus (Ut) indicated. (B) Labeling of the abdominal ganglion shown as a confocal stack. The white arrow in panel (B) indicates the specific labeling of a yellow cell, allowing an unambiguous match to yellow processes in the reproductive tract. This cell is indicated as SpA in Figure 4. The white arrowhead and asterisk indicate two cells that are similarly labeled red and therefore cannot be assigned to processes in the reproductive tract based on this preparation alone. Based on other labelings, the larger more posterior cell is PC1 and the smaller, anterior cell is SpB. At least one large blue cell body is labeled (double white arrowheads), but additional, smaller blue cell bodes appear to be labeled blue in this preparation. Based on other labelings, the large blue cell is PC4, which innervates the ovaries. Neuropil anterior to the posterior cluster is labeled “Ant Npl”. Panels (C,D,E) correspond to boxed areas in panel (A) and include the stalk of the spermatheca (C), the anterior uterus (D), and the ovaries (E). Insets in panels (C–E) represent single optical sections of the confocal stack shown in panel (B). (C) Yellow processes correspond to the yellow cell (SpA) in both the inset and panel (B) (white arrows). On the basis of other labeling experiments, the red processes (asterisks) correspond to the small red cell labeled with an asterisk in panel B (SpB in Figure 4). (D) On the basis of other labelings, the red processes in the uterus and posterior oviduct are derived from the indicated red cell (white arrowhead in inset) and represent PC1. (E) The blue processes correspond to large blue cell innervating the ovaries (white double arrowheads in inset and in panel B) and is indicated as PC4 in Figure 4. Scale bars: (A,C–E): 50 μm. (B) and insets in (C,D,E): 10 μm. [file Image_1.tif]

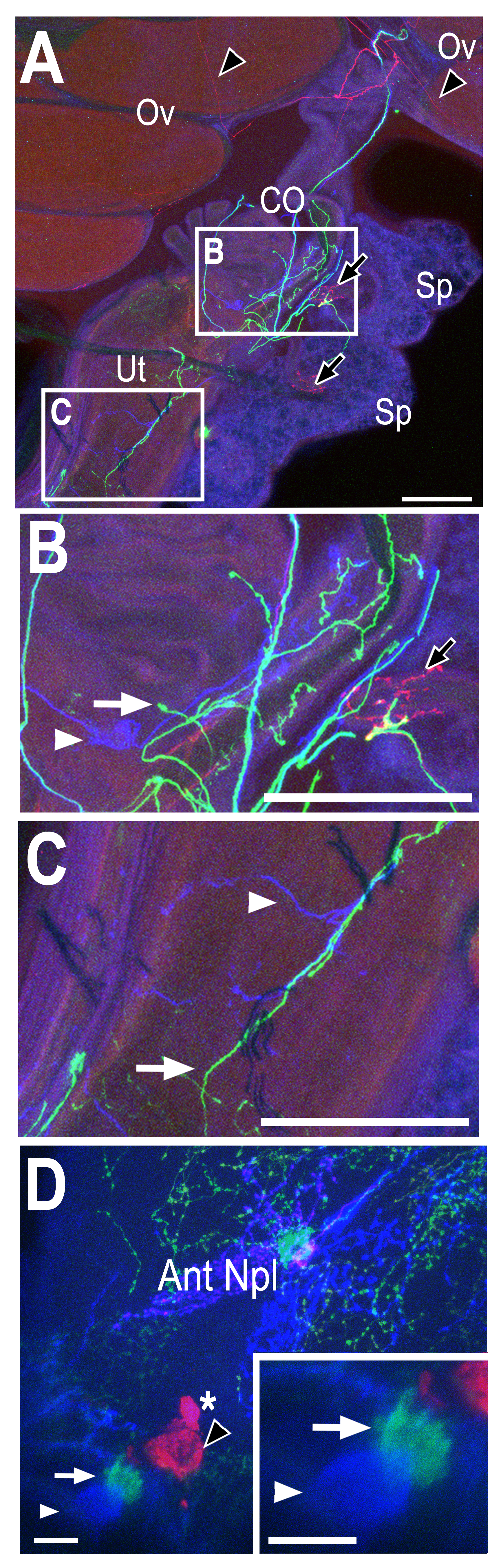

Supplement: SUPPLEMENTARY FIGURE S2 — Two neurons innervate the posterior common oviduct and uterus. (A) Overview of the labeled reproductive tract with the ovaries (Ov), common oviduct, (CO), spermathecae (Sp), and uterus (Ut) indicated. Red processes in the ovaries (black arrowheads) and spermathecae (black arrows) are also indicated. (B,C) The boxed regions from panel (A) shown at higher magnification include the posterior common oviduct (B) and the uterus (C) with blue (white arrowhead), green (white arrow) and red (black arrow) processes indicated. (D) Labeled cells in the abdominal ganglion include one blue (white arrowhead) and one green (white arrow) cell represented as PC1 and PC2 in Figure 4, respectively. Two red cells are visible. Based on a comparison to other labelings, the smaller red cell (asterisk) and the larger red cell (black arrowhead) innervate the stalk of the spermathecae and the ovaries respectively and are indicated as SpB and PC4 in Figure 4. Scale bars: (A): 50 μm. (B–D) and inset in (D):10 μm. [file Image_2.tif]

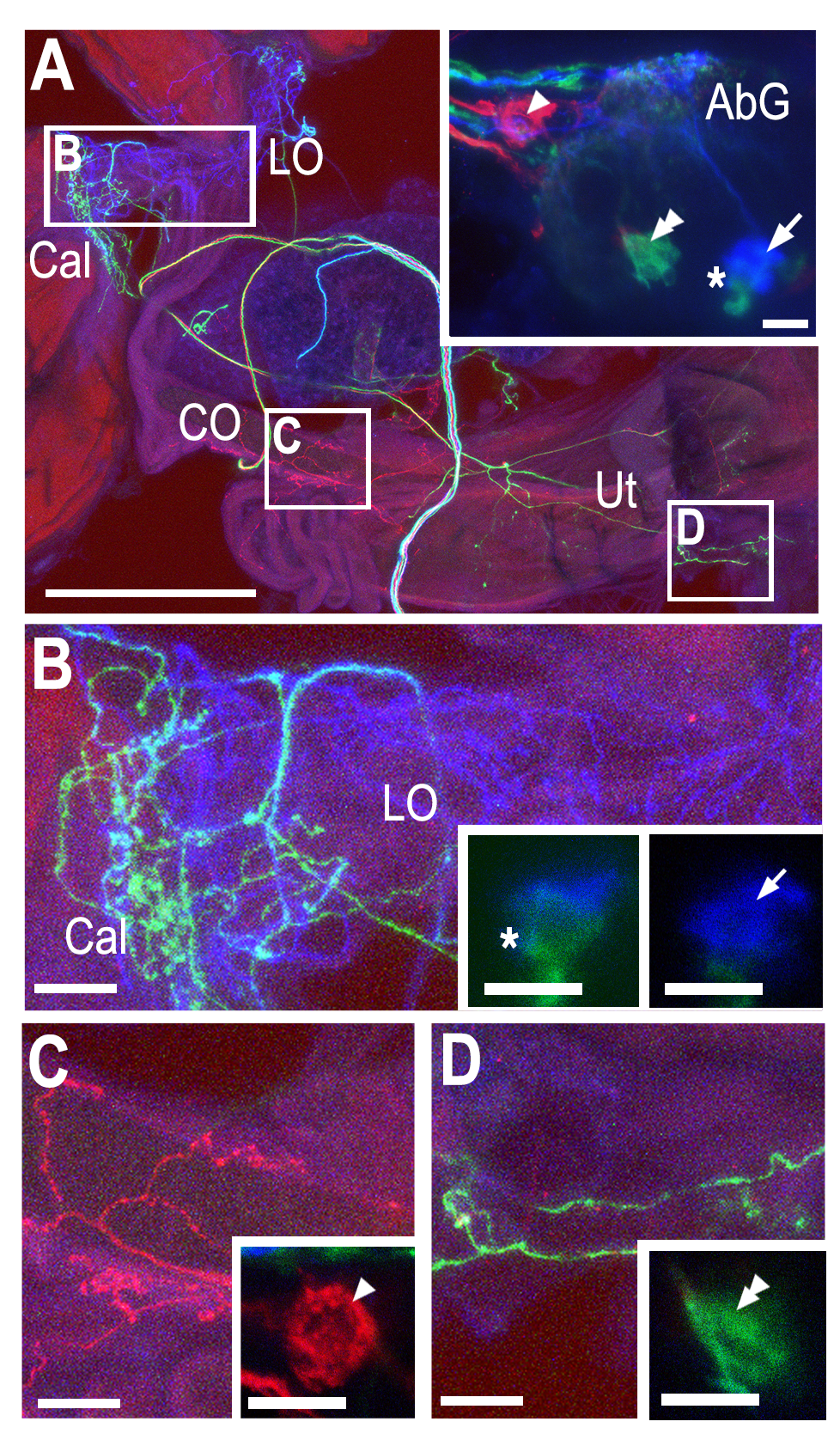

Supplement: SUPPLEMENTARY FIGURE S3 — A neuron innervating the posterior uterus. (A) Overview of the labeled reproductive tract with the calyx (Cal), lateral oviduct (LO), common oviduct (CO) and uterus (Ut) indicated. The panel (A) inset shows labeling of the abdominal ganglion (AbG) as a confocal stack and includes one red cell (single white arrowhead), one blue cell (white arrow), and two green cells (asterisk and double white arrowhead). (B–D) Higher magnification of the boxed regions in panel (A). Insets in panels (B–D) show single optical sections of cells indicated in the panel (A) inset. Identification of the red cell as PC1 (projecting to the posterior oviduct and anterior uterus) and the blue cell as PC6 (projecting to the lateral oviduct and calyx) can be determined from this preparation alone since only one red cell and one blue cell were observed in the abdominal ganglion. Comparison of this preparation to other labelings allows assignment of the small green cell (asterisk) as PC7 (projecting to the calyx), and the larger green cell as PC3 (double white arrowheads, projecting to the posterior uterus). Scale bars: (A): 100 μm. (B–D) and insets in (A–D): 10 μm. [file Image_3.tif]
