# Supplementary material for: Heterogeneity in the projections and excitability of tyraminergic/octopaminergic neurons that innervate the Drosophila reproductive tract
Source: Front Mol Neurosci. 2024 Aug 2;17:1374896. doi: 10.3389/fnmol.2024.1374896 (PMC11327148; doi:10.3389/fnmol.2024.1374896)
Supplement: Supplementary file 4 [file Table_1.docx]

**Supplemental Table T1. Summary of statistical analysis.** For each of the indicated data sets, the distribution, statistical test used, effect size and adjusted p value are listed.

| **Data** | **Data Distribution** | **Test** | **Power** | **Adjusted p value** |
| --- | --- | --- | --- | --- |
| Optogen. (Fig 6)  Stim 1  3 gp. comp. | Non-Gaussian | Kruskal-Wallis | Mean rank difference  Cont. vs Tdc2: -13.44  Cont. vs J39942: -11.09  Tdc2 vs J39942: 2.34 | 0.0015  0.0060  0.99999 (ns) |
| Optogen. (Fig 6)  Stim 2  3 gp. comp. | Non-Gaussian | Kruskal-Wallis | Mean rank difference  Cont. vs Tdc2: -14.25  Cont. vs J39942: -11.73  Tdc2 vs J39942: 2.52 | 0.0008  0.0036  0.99999 (ns) |
| Optogen. (Fig 6)  Stim 1 freq. | Non-Gaussian | Mann-Whitney | Diff. between medians:  0.15 | 0.225 (ns) |
| Optogen. (Fig 6)  Stim 2 freq. | Non-Gaussian | Mann-Whitney | Diff. between medians:  -0.003 | 0.76 (ns) |
| Optogen. (Fig 6)  Stim 1 latency | Non-Gaussian | Mann-Whitney | Diff. between medians:  -2.5 | 0.117 (ns) |
| Optogen. (Fig 6)  Stim 2 latency | Non-Gaussian | Mann-Whitney | Diff. between medians:  -1.0 | 0.81 (ns) |
| Ephys PC1 vs PC2 (Fig 7) | Non-Gaussian | Linear regression | Cell type coefficient  Estimate: -5.72  95% CI: -6.67 to -4.78 | <2 x 10^-16^ |
| Ephys PC1  drugs  (Fig 8E) | Non-Gaussian | Linear regression | Ivermectin coefficient  Estimate: -0.089  95% CI: -0.169 to -0.009  PTx coefficient  Estimate: 0.296  95% CI: 0.213 to 0.379 | 0.029  2.67 x 10^-11^ |
| Ephys PC2  drugs  (Fig 8F) | Non-Gaussian | Linear regression | Ivermectin coefficient  Estimate: 0.262  95% CI: 0.202 to 0.321  PTx coefficient  Estimate: 0.316  95% CI: 0.265 to 0.367 | <2 x 10^-16^  <2 x 10^-16^ |
