# Supplementary material for: Heterogeneity in the projections and excitability of tyraminergic/octopaminergic neurons that innervate the Drosophila reproductive tract
Source: Front Mol Neurosci. 2024 Aug 2;17:1374896. doi: 10.3389/fnmol.2024.1374896 (PMC11327148; doi:10.3389/fnmol.2024.1374896)
Supplement: Supplementary file 5 [file Table_2.docx]

**Supplemental Table T2. Reagent sources.** The source, reference number and RRID (if available) are listed for each of the biochemical and genetic reagents used in this study.

| **Reagent** | **Expt.** | **Conc** | **Source** | **Ref#** | **RRID** |
| --- | --- | --- | --- | --- | --- |
| Mouse-anti-V5 (monoclonal) | MCFO | 1/500 | ThermoFisher | MA5-15253 | AB_10977225 |
| Rabbit-anti-ΗA (monoclonal) | MCFO | 1/300 | Cell Signaling Technology, Danvers, MA | 3724 | AB_1549585 |
| Rat-anti-FLAG (monoclonal) | MCFO | 1/200 | Novus Biologicals | NBP1-06712 | AB_1625981 |
| Goat anti-Mouse-  Alexa Fluor488 | MCFO  IHC | 1/500 | ThermoFisher | A-32723 | AB_2633275 |
| Goat anti-Rabbit-  Alexa Fluor 555 | MCFO  IHC | 1/500 | ThermoFisher | A-21428 | AB_2535849 |
| Goat anti-Rat-AlexaFluro633 | MCFO | 1/500 | ThermoFisher | **A-21094** | AB_2535749 |
| Mouse anti-GFP (monoclonal) | IHC | 1/500 | Sigma-Aldrich | G6539 | AB_259941 |
| Rabbit anti-dsRED | IHC | 1/500 | Takara | 632496 | AB_10013483 |
| Streptavidin-  AlexaFluor555 | ephys |  | ThermoFisher | S-21381 |  |
| Biocytin | ephys |  | Sigma-Aldrich | B1758 |  |
| Picrotoxin | ephys |  | Sigma-Aldrich | P8390 |  |
| Ivermectin | ephys |  | Sigma-Aldrich | I8898 |  |
| Fluoromount-G | MCFO  IHC |  | SouthernBiotech | 0100-01 |  |
| DAPI | IHC |  | Sigma-Aldrich | D9542 |  |
| AF555-conjugated Phalloidin | IHC | 1/500 | Invitrogen | A34055 |  |
| *UAS-MCFO-7* | MCFO |  | Bloomington *Drosophila* stock center | 64091 |  |
| *Tdc2-Gal4* | Optogenet |  | Bloomington *Drosophila* stock center | 9313 |  |
| *UAS-ChR2XXM-TdTomato* | Optogenet |  | Dr. Robert Kittel  (University of  Würzberg) | --- |  |
| *LexAop::CD2-RFP, UAS::mCD8-GFP* | IHC |  | Bloomington *Drosophila* stock center | 67093 |  |
| *J399342-Gal4* | IHC  Optogenet |  | Bloomington *Drosophila* stock center | 39942 |  |
| *Tdc2-LexA* | IHC |  | Bloomington *Drosophila* stock center | 52242 |  |
| *GluCl-MiMIC-T2A-Gal4* | IHC |  | Bloomington *Drosophila* stock center | 77841 |  |
